# Redundant and distinct mechanisms suppress innate immune activation during SARS-CoV-2 infection

**DOI:** 10.1371/journal.pbio.3003808

**Published:** 2026-05-20

**Authors:** Fuchun Zhou, Sivakumar Periasamy, Nathaniel D. Jackson, Wan Sze Cheng, Ruben Soto Acosta, Aarti Tripathi, Kritika Kedarinath, Philipp A. Ilinykh, Chengjin Ye, Shailendra Chauhan, German Nudelman, Elena Zaslavsky, Haiping Hao, Steven G. Widen, Luis Martinez-Sobrido, Stuart C. Sealfon, Alexander Bukreyev

**Affiliations:** 1 Department of Pathology, University of Texas Medical Branch, Galveston, Texas, United States of America‌‌; 2 Galveston National Laboratory, Galveston, Texas, United States of America; 3 Department of Disease Intervention and Prevention, Texas Biomedical Research Institute, San Antonio, Texas, United States of America‌‌; 4 Department of Neurology, Icahn School of Medicine at Mount Sinai, New York, New York, United States of America; 5 Department of Biochemistry and Molecular Biology, University of Texas Medical Branch, Galveston, Texas, United States of America; 6 Department of Microbiology and Immunology, University of Texas Medical Branch, Galveston, Texas, United States of America; 7 Center for Biodefense and Emerging Viral Infections, University of Texas Medical Branch, Galveston, Texas, United States of America; Ulm University Medical Center, GERMANY

## Abstract

Several SARS-CoV-2 proteins have been shown to counteract the host innate immune response, mostly using in vitro protein expression, which may not fully reflect their role in the context of viral infection. In addition, while each viral protein was characterized in a different experimental system, its relative contribution to immunosuppression remains unclear. Here we used a SARS-CoV-2 bacterial artificial chromosome with *en* *passant* mutagenesis to recover a panel of 12 infectious recombinant SARS-CoV-2 viruses, each with mutations in either NSP1, NSP2, NSP3, NSP6, NSP12, NSP13, NSP14, NSP15, NSP16, ORF3a, ORF6, or ORF8. We used the interferon-stimulated response element (ISRE)-driven luciferase assay in 293T-ACE2/TMPRSS2 cells to test the panel, demonstrating that mutations in many proteins, especially in NSP1 and NSP15, increased the type I interferon response relative to the parental wild-type virus. RNA-seq analysis of mutant-virus infected Calu-3 cells showed that the mutations in NSP1 or NSP15 lead to higher expression of multiple genes involved in innate immune response, cytokine-mediated signaling, and regulation of lymphocyte proliferation. Furthermore, mutations in either NSP1 or NSP15 resulted in a greater maturation of human monocyte-derived dendritic cells in vitro. Infection of K18 hACE2 transgenic mice with either NSP1 or NSP15 mutated viruses demonstrated attenuated respiratory tract replication. Analysis of lung immune cells from infected mice by single-cell RNA-seq identified 15 populations of major myeloid and lymphoid cells with changes in the pattern of their activation associated with viral infection. The effects of mutations in NSP1 or NSP15 on these responses are consistent with differences in the immunosuppressive mechanisms utilized by the two proteins. Overall, these data demonstrate different and redundant mechanisms of innate immune antagonism by SARS-CoV-2 and suppression of activation of antigen-presenting cells and T and B lymphocytes mediated by multiple viral proteins.

## Author summary

The mechanisms by which SARS-CoV-2 and its proteins modulate host immunity, specifically the interferon response, are still not clear. We generated 12 infectious SARS-CoV-2 viruses with mutations in individual proteins and demonstrated that many of them have interferon-antagonizing activity and immunosuppressive effects in human cells and in the K18 hACE mouse model of infection. We identified distinct and redundant mechanisms of immunosuppression of SARS-CoV-2 mediated by multiple individual viral proteins, with 9 out of the 12 tested proteins showing some immunosuppressive effect in at least one experimental system. The demonstrated immunosuppressive effects extend from the innate response to immune cells to pathologic changes in vivo. Importantly, this work shows, for the first time, a comparison of the effects of multiple viral proteins in the context of authentic viral infection, rather than in a surrogate system, and shows the relative contribution of each viral protein under identical experimental conditions. Overall, our data indicates that SARS-CoV-2 antagonizes multiple immune mechanisms, particularly type I interferon signaling, activation of innate immune cells and T and B lymphocyte functions, with the greatest effects due to NSP1 and NSP15.

## Abstract 3

SARS-CoV-2 suppresses innate immunity through multiple proteins, but their relative roles during infection remain unclear. This study characterizes the role of a panel of 12 viral proteins, showing that they, especially NSP1 and NSP15, act through distinct yet redundant mechanisms to inhibit interferon responses and immune cell activation.

## Introduction

The COVID-19 pandemic, caused by SARS-CoV-2, has caused more than 776 million confirmed cases of human disease, including more than 7 million deaths (October 2024) [1]. Since the first report on the occurrence and rise of COVID-19 cases, SARS-CoV-2 has shown a powerful transmission potential that has contributed to the deadly pandemic. This virus has continuously evolved into variants of concern (VOC) with a higher ability for transmission and continuing the pandemic beyond 2 years [2]. SARS-CoV-2, similarly to SARS-CoV, enters mammalian cells using the angiotensin-converting enzyme 2 (ACE2) receptor, which is abundant in the respiratory and intestinal epithelial cells [3]. The virus causes diffuse alveolar damage and COVID-19 associated acute respiratory distress syndrome (ARDS) through dysfunctional immune responses [4]. In addition, it also causes acute tissue injury, particularly in the liver and kidneys, coagulopathies (disseminated intravascular coagulation and fibrin thrombi formation), thrombocytopathy, and pulmonary embolism [4]. The large RNA genome of SARS-CoV-2 contains multiple open reading frames (ORFs) which encode 4 structural proteins (S, E, M, and N), 16 nonstructural proteins (NSP1-16), derived from ORF1a and ORF1b polyproteins, and several accessory proteins, including ORF3a, ORF3b, ORF3c, ORF3d, ORF6, ORF7a, ORF7b, ORF8, ORF9b, ORF9c, and ORF10. The 16 NSPs are from the processing of the polyprotein precursors by the viral NSP3 and NSP5, which have the activity of papain-like protease (PLpro) and the 3C-like protease (3CLpro), respectively. NSP3 is responsible for the proteolytic cleavage of NSP1-4, and NSP5 for the processing of other cleavage sites that result in NSP5-16 [5]. While NSPs play a critical role in viral replication, the accessory proteins do not, but both groups of proteins play an important role in viral pathogenesis and modulating the host immune response [6].

Interferons (IFN) are important secreted host proteins with strong anti-viral functions. Type I IFNs (IFN-I), which include IFNα and IFNβ, are produced by many cells in response to viral infections, including SARS-CoV-2 [7]. Data from clinical patients indicate that low induction of IFN-I at the local and systemic levels correlates with the severity of COVID-19 [8–10]. In some patients with severe COVID-19, high nasal viral titers correlate with low IFN-I levels, and in some cases with high titers of autoantibodies against IFNs. This suggests that an appropriate induction of high titers of IFNs enables epithelial cells to inhibit SARS-CoV-2 replication and growth at nasopharyngeal and other mucosal sites [11]. However, SARS-CoV-2 antagonizes the innate host defense mechanisms, including the inhibition of IFN-I production and signaling [12], allowing the virus to replicate exponentially, causing severe tissue pathologies.

Multiple coronavirus NSPs and accessory proteins antagonize IFN-I signaling (Table 1). Specifically, SARS-CoV-2 NSP1, NSP2, NSP3, NSP5, NSP6, NSP8, NSP9, NSP12, NSP13, NSP14, NSP15, NSP16, and accessory proteins ORF3a, ORF6, ORF8 modulate host innate immune response at several levels, including disruption of host RNA splicing and translation, interference of protein trafficking and modification of host protein-protein interactions [13–46]. Most of the immune antagonism studies described above relied in vitro cell cultures transfected with plasmids expressing SARS-CoV-2 proteins [13–24,26,29,30,36,38,41,43–46] and some used purified recombinant proteins [14,28,31,32,42]. However, this system most likely does not recapitulate the biological effects of the proteins in the context of authentic SARS-CoV-2 infection. Importantly, the previous studies characterized the effects of innate immune-antagonizing viral proteins individually and in different experimental systems, making assessment of their relative importance impossible.

**Table 1:** The selected proteins, mutations, and targeted domains or amino acids.

Mutant number	Mutated gene	Mutation	Was virus recoverable?	Known role of the protein related to IFN-I antagonism	Known role of the targeted domain or amino acid	References
M1	NSP1	K164A/H165A	Yes	Reduces expression of host innate immune sensors and IFN-I signaling	Binds to the 40S ribosomal subunit	[14,19]
M2	NSP2	Q321A	Yes	Inhibits IFN-I production	May confer higher stability to the protein	[25,47]
M3	NSP3	C856A	No	Regulates IFN-I pathway	Essential catalytic cysteine in the PLpro domain involved in cleavage of the viral polyprotein	[39,48]
M4	NSP3	N901A	Yes	Regulates IFN-I pathway	Contributes to deubiquitnase/deISGylase activity	[48,49]
M5	NSP3	R911S/E912R	No	Regulates IFN-I pathway	Contributes to deubiquitnase/deISGylase activity	[48,49]
M6	NSP5	C145A	No	Inhibits IFN-I induction	Essential for catalytic function of NSP5	[50,51]
M7	NSP6	AA268–279 deletion	No	Suppresses IFN-I production	Contribute to membrane remodeling and replication organelle formation, helps recruit host factors that support replication	[13,52]
M8	NSP6	L37F	Yes	Suppresses IFN-I production	Mutation L37F disrupts the folding stability of NSP6	[13,53]
M9	NSP12	F480L	Yes	Attenuates IFN-I production	Stabilizes the overall polymerase fold	[26,54]
M10	NSP13	A336V	Yes	Inhibits IFN-I production	Contributes to helicase and ATPase activities	[29,55]
M11	NSP14	D352A	Yes	Blocks IFN-I signaling	The residue is conserved and is critical for N7-MTase enzymatic activity	[40,56]
M12	NSP15	H234A	Yes	Suppresses IFN-I production	A catalytic residue in the NSP15 EndoU active site	[31,41]
M13	NSP16	D130A	Yes	Suppresses IFN-I response	Contributes to 2′-O-methyltransferase activity	[42]
M14	ORF3	ORF3a (AA1-275) deletion	Yes	Antagonizes IFN-I signaling	Inhibits interferon-activated JAK/STAT signaling	[43]
M15	ORF6	Change AA53-61 (DEEQPMEID) to AAAAAAAAA	Yes	Inhibits IFN-I production and downstream signaling	Suppresses IRF3 activation	[21]
M16	ORF8	AA51-56 (ARKSAP) deletion	Yes	Inhibits IFN-I induction and signaling	The ARKSAP motif mimic host histone and alters chromatin and host gene expression	[44,57]

Here, we investigated inhibition of IFN-I signaling by viral proteins in the context of replication-competent SARS-CoV-2. We generated 12 infectious SARS-CoV-2 viruses with mutations in individual proteins and demonstrated that many of them have IFN-I-antagonizing activity and immunosuppressive effects in human cells and in the K18 hACE mouse model of infection. Our data indicate that SARS-CoV-2 antagonizes multiple immune mechanisms, particularly IFN-I signaling, activation of innate immune cells and T and B lymphocyte functions, with the greatest effects due to NSP1 and NSP15.

## Results

### Generation of SARS-CoV-2 mutant viruses

**Fig 1:**
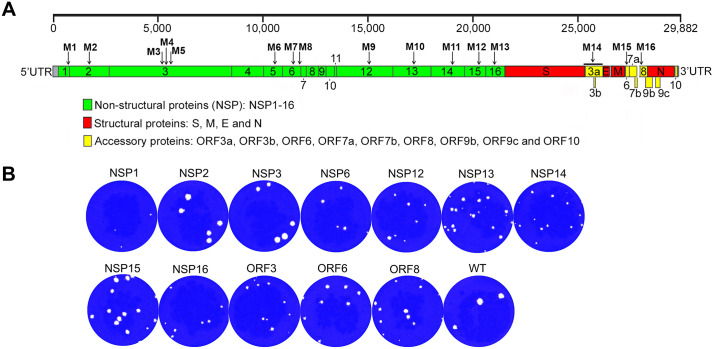
Generation of SARS-CoV-2 mutant strains. **A.** Schematic representation of the SARS-CoV-2 genome. The positions of the mutations are indicated by arrows with mutation numbers. **B.** Comparison of plaques of WT SARS-CoV-2 and the mutants in Vero E6 cells on day 2 post infection.

To investigate the contribution of SARS-CoV-2 proteins or domains on viral pathogenesis and protective immune mechanisms, we selected 13 genes and specific mutations based on the available literature on several coronaviruses [13,16,19,23,25,26,29,30,32,45,50,58,59] (Table 1). To generate SARS-CoV-2 mutants, we first introduced mutations into the viral genes in an infectious cDNA clone of strain USA-WA1/2020 pBeloBAC11-SARS-CoV-2 [60], which was driven by the human cytomegalovirus immediate early gene promoter, using *en passant* mutagenesis [61] (the primers for mutagenesis are shown in S1 Table). After the mutations were confirmed in the cDNA clones by Sanger DNA sequencing, Vero E6 cells were transfected with the cDNA clones containing the desired mutations for recombinant virus recovery. We generated a total of 16 mutated cDNA clones (Table 1, Fig 1A). To recover viruses from the mutated cDNA clones, 80% confluent monolayers of Vero E6 cells in 12-well plates were transfected with 1.0 μg per well of infectious SARS-CoV-2 BAC DNA WT or its mutated derivatives. At 48 h post-transfection, transfected cells were split and seeded into 25 cm^2^ flasks with 50% confluent monolayers of Vero E6 cells. The cell cultures were observed daily until the appearance of cytopathic effect to collect viral supernatants. Viruses were recovered from the wild-type (WT) nonmutated control and the 12 cDNA clones containing mutations in NSP1, NSP2, NSP3, NSP6, NSP12, NSP13, NSP14, NSP15, NSP16, ORF3a, ORF6, or ORF8 (Table 1). The recovered viruses were expanded by an additional 1 or 2 passages, and the viral genomic RNA was isolated from the cell culture supernatants. Sequencing of the viral genomes confirmed the expected mutations (S1 Fig). We could not recover viruses from the cDNA clones carrying two separate mutations in NSP3, or a mutation in NSP5, or a deletion in NSP6 (Table 1). All the viruses were titrated in Vero E6 cells by plaque assay, and their plaque sizes were compared. We found that all the mutant viruses, except the NSP2 and NSP3 mutants, have a smaller plaque phenotype when compared to the WT virus (Fig 1B). Growth kinetics experiment in human epithelial Calu-3 cells demonstrated the most pronounced attenuation of the NSP1, NSP6, NSP14, NSP16, ORF3, and ORF6 mutants (S2A Fig). Generally, attenuation of replication corresponded to reduced plaque phenotype (Fig 1B) with one notable exception. The NSP12 mutant demonstrated reduced plaque phenotype yet WT-like replication. We also compared replication of the NSP1 and NSP15 mutants selected for in-depth investigation (below) with that of WT virus in Vero-AT cells, which express human angiotensin receptor 2 (hACE-2) and transmembrane serine protease 2 (TMPRSS2), and A549-hACE2 cells, which express hACE-2, that demonstrated their attenuation (S2B Fig). The attenuation of the NSP1 and NSP15 mutants in Vero-AT cells, which are deficient in IFN-I production [62], is consistent with the competence of Vero cells in type III interferon production [63].

### Multiple SARS-CoV-2 NSP proteins contribute to inhibition of IFN-I induction

**Fig 2:**
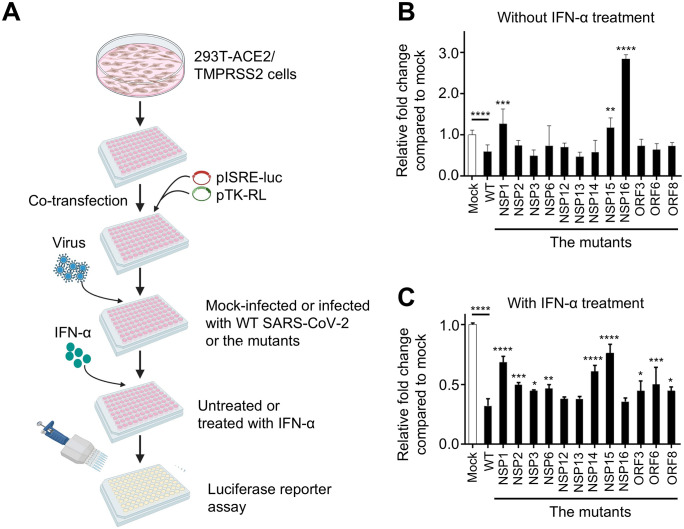
SARS-CoV-2 NSP1 and NSP15 are the most potent antagonists of IFN-I response. **A.** Flowchart of the experiment. 293T-ACE2/TMPRSS2 cells were transfected with pISRE-luc and pTK-RL. At 24 h after the transfection, the cells were mock-infected or infected with WT SARS-CoV-2 or the 12 mutants. At 24 h after the infection, cells were mock-treated or treated with IFN-α for an additional 24 h, and the luciferase activity of the cell lysates was measured. Created in BioRender. Zhou, F. (2026) https://BioRender.com/x94u104. **B, C.** Relative fold change compared to mock-infected cells in luciferase activity for WT and mutated viruses in 293T-ACE2/TMPRSS2 cells without (B) and with (C) IFN-α treatment. Mock, uninfected cells. Mean values ± SD based on triplicate samples. Significance of differences for each mutant vs. WT are shown by asterisks and separately shown for WT compared to mock-infected cells (Mock). The significance was determined by one way ANOVA. * *p* < 0.05, ** *p* < 0.01, *** < 0.001, **** *p* < 0.0001. The data underlying panels B and C in this Figure can be found in S1 Data.

Using the panel of 12 mutated SARS-CoV-2, we investigated the impact of NSP and ORF proteins on IFN-I signaling. We transfected 293T-ACE2/TMPRSS2 cells, constitutively expressing SARS-CoV-2 receptors ACE-2 and TMPRSS2 (ACE2/TMPRSS2) [64], with pISRE-luc firefly luciferase reporter plasmid and pTK-RL renilla luciferase reporter plasmid for control of transfection efficiency. The next day, cells were mock-infected (phosphate buffered saline, PBS) or infected with SARS-CoV-2 WT or mutant viruses at 0.3 plaque-forming units (PFU) per cell. After 24 h of infection, cells were either left untreated or treated with IFN-α (100 U/ml) and incubated for an additional 24 h, and then the ISRE-induced luciferase activity was measured (Fig 2A). As expected, WT SARS-CoV-2 inhibited the ISRE-luc activity when compared to mock-infected cells, presumably due to the combined effect of multiple IFN-I-antagonizing proteins of SARS-CoV-2 (Fig 2B). Importantly, significantly higher ISRE-luc activities were observed in cells infected with NSP1, NSP15, and NSP16 mutant viruses as compared to WT SARS-CoV-2 (Fig 2B). Even with IFN-α treatment, WT SARS-CoV-2 inhibited ISRE-luc activity (Fig 2C). In contrast, most of the mutant viruses – NSP1, NSP2, NSP3, NSP6, NSP14, NSP15, ORF3a, ORF6, and ORF8 – showed higher ISRE-luc activity than WT SARS-CoV-2.

To determine if the differences in IFN-I antagonism between the viruses were related to different rates of infection, we infected 293T-ACE2/TMPRSS2 cells with the WT or mutated viruses in triplicate, incubated the monolayers for 24 h, and stained the nuclei with DAPI and the infected cells with NSP3-specfic antibodies. Calculation of the infection rates demonstrated no difference between the percentages of infected cells (S3 Fig), suggesting that the increased IFN-I antagonism detected for some mutants is not due to the change in the rate of infection. Overall, these data suggest multiple and redundant mechanisms of IFN-I antagonism mediated by SARS-CoV-2, with the major effects mediated by NSP1, NSP15, and NSP16, and lesser effects mediated by the other viral proteins.

### SARS-CoV-2 NSP1 and NSP15 suppress maturation of human dendritic cells

**Fig 3:**
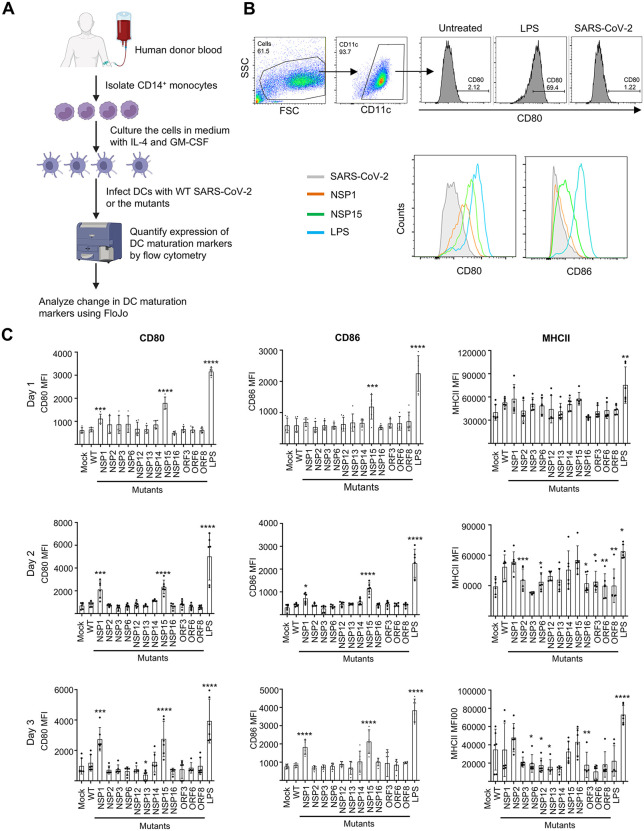
SARS-CoV-2 NSP1 and NSP15 suppress maturation of human dendritic cells. **A.** Flowchart of the experiment. CD14+ monocytes were isolated from fresh human PBMCs and further cultured in the presence of IL-4 and GM-CSF for differentiation into DCs. On day 6, the DCs were treated with LPS, or mock-infected or infected with WT SARS-CoV-2 or the 12 mutants at MOI of 3 PFU/cell, cultured for 1, 2, or 3 days and analyzed for expression of maturation markers by flow cytometry. Created in BioRender. Zhou, F. (2026) https://BioRender.com/z01a576. **B.** Top: gating strategy with representative samples cultured for 2 days. Bottom: histograms showing expression of maturation markers CD80 and CD86 in representative samples cultured for 2 days. **C.** MFI of the DC maturation markers CD80, CD86 and also MHCII in CD11c+ DCs at day 1 (top panel), day 2 (middle panel), and day 3 (bottom panel). Data represent mean ±SD from three independent healthy donors, each analyzed in the two technical replicates. The significance of differences for each mutant vs. WT was determined by one way ANOVA: *<0.05, **<0.01, ***<0.001, ****0.0001. The data underlying panel C this Figure can be found in S1 Data.

SARS-CoV-2 modulates the function of T cells, natural killer cells, monocytes, and specifically impairs the functional phenotypes of dendritic cells (DCs) [65–67]. As noted above, SARS-CoV-2 antagonizes IFN-I production and signaling. IFN-I promotes the phenotypic and functional maturation of DCs [68–70]. Experimental infection of human monocyte-derived DCs results in abortive infection with abundant production of the viral proteins and antiviral and proinflammatory cytokines [71]. We therefore tested the effects of SARS-CoV-2 WT and the mutants on the maturation of immature human monocyte-derived DCs. Cells were stimulated with lipopolysaccharide (LPS, positive control), SARS-CoV-2, WT, or its mutants for 1, 2, or 3 days (Fig 3A). The maturation status of CD11c+ DCs was assessed by flow cytometry, measuring the expression of maturation markers CD80 and CD86, with HLA-DR/MHCII also assessed as a reference marker. As expected, LPS treatment significantly up-regulated the expression of CD80 and CD86 at all three timepoints, as seen in mean fluorescent intensity (MFI) (Fig 3B, 3C). However, SARS-CoV-2 showed no DC maturation based on the induction of the two markers, which was comparable to mock-infected DCs (Fig 3C). Strikingly, the NSP1 and NSP15 mutant viruses induced the maturation of the two markers at a significantly higher level than WT SARS-CoV-2. These effects are even more important in the context of the modest yet significant attenuation of these viruses (S2 Fig). In contrast, NSP13 reduced CD80 expression at day 3, which is a deviation from other mutants that did not alter the expression of the maturation markers. Although MHCII expression was noted in most of the cells, its MFI was highly variable depending on the stimulants (Fig 3C). Together, these data indicate that SARS-CoV-2 suppresses DC maturation, and that the effect is mainly mediated by NSP1 and NSP15.

### SARS-CoV-2 NSP1 and NSP15 suppress genes that control innate immune response

**Fig 4:**
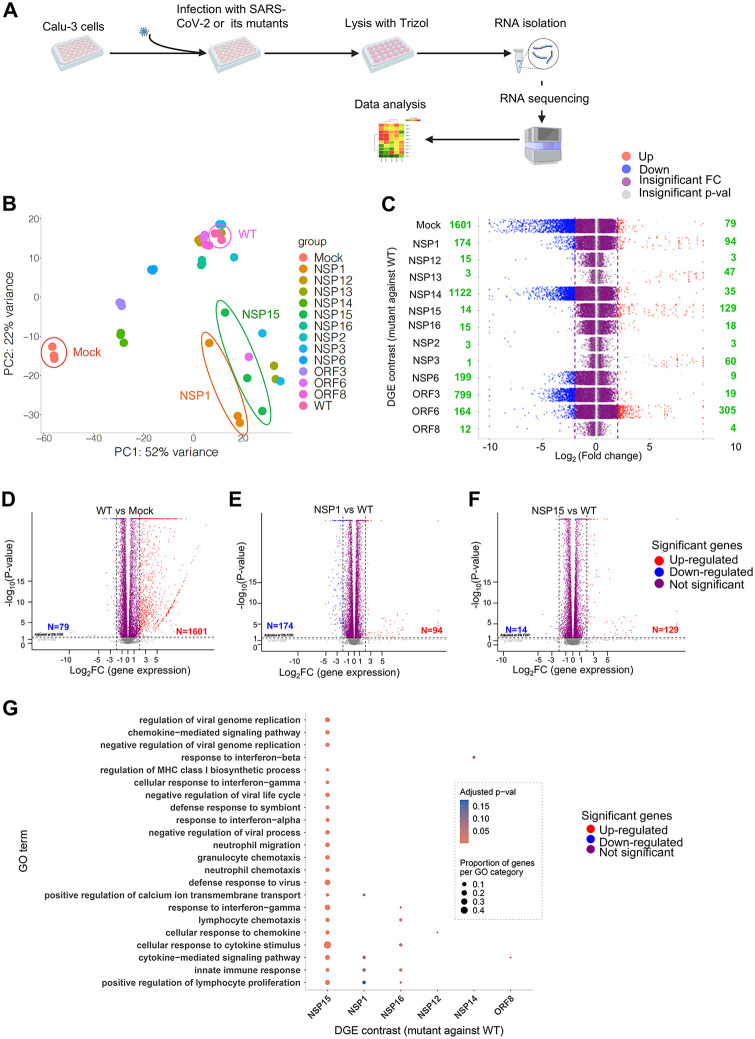
SARS-CoV-2 NSP1 and NSP15 suppress expression of genes involved in mechanisms of cell defense. **A.** Flowchart of the experiment. Calu-3 cells were mock-infected or infected in triplicates with WT SARS-CoV-2 or the 12 mutants, and 24 h later, the cells were harvested in Trizol for RNA isolation, followed by RNA sequencing and data analysis. Created in BioRender. Zhou, F. (2026) https://BioRender.com/j86r787. **B.** Principal component analysis (PCA) to identify the distance or clusters of gene activation by WT SARS-CoV-2 or the 12 mutants. **C.** Jitter plot analysis for change in differential gene expression (DGE) in Calu-3 cells mock-infected or infected with the 12 SARS-CoV-2 mutants: comparison with WT SARS-CoV-2-infected cells. The number of up- and down-regulated genes is indicated in green. **D.** Volcano plot for DGE in WT vs. mock. 1,601 genes were up-regulated (Log_2_FC > 2) and 79 genes were down-regulated (Log_2_FC > 2) in WT SARS-CoV-2-infected Calu-3 cells compared with mock-infected cells. **E.** Volcano plot for DGE in NSP1 vs. WT. Ninety-four genes were up-regulated (Log_2_FC > 2) and 174 genes were down-regulated (Log_2_FC > 2) in NSP1 mutant-infected Calu-3 cells compared with mock-infected cells. **F.** Volcano plot for DGE in NSP15 vs. WT. 129 genes were up-regulated (Log_2_FC > 2) and 14 genes were down-regulated (Log_2_FC > 2) in NSP15 mutant-infected Calu-3 cells compared with mock-infected cells. **G.** Gene Ontology (GO) term analysis showing pathway enrichment for NSP1 and NSP15 over WT. NSP1, NSP15 inhibit several pathways, including innate immune response and cytokine-mediate signaling pathways. The data underlying this Figure can be found in GEO database, accession number GSE254699.

To explore the effects of SARS-CoV-2 proteins on expression of host genes, we first mock-infected (PBS) or infected Calu-3 cells with MOI of 1.5 PFU per cell with SARS-CoV-2 WT or the 12 mutants. At 24 h post-infection the cells were used for the isolation of RNA, which was deep sequenced (Fig 4A). Principal component analysis (PCA) of transcriptome from 42 samples (3 repeats for each virus and mock infections) demonstrated that gene expression patterns in the cells infected with SARS-CoV-2 WT and the 12 mutants were different from those in mock-infected cells and from each other (Fig 4B). The differential expression analysis demonstrated that the mutants up- or downregulated cellular gene expression to a different extent. NSP3, NSP13, and NSP15 mutants upregulated more genes, while the NSP6, NSP14, and ORF3a mutants downregulated more genes when compared to the WT virus. However, the NSP1 and ORF6 mutants both were able to up- and down-regulate quite different sets of host genes (Fig 4C).

Infection of Calu-3 cells with WT SARS-CoV-2 up-regulated more than 1,500 genes compared to mock-infected cells (Fig 4D). The upregulated genes included those involved in innate immune response and are known responders of IFN-I stimulation [72]: *IFNB1, CXCL10, OAS2, TNFAIP3 RSAD2, IFNL2, USP18, MX1, CXCL11, NFKBIA, IFIT2, ATF3, IFIT1, IFNL3,* and *IFI44L*. Among these, OAS2 is an important antiviral protein involved in innate immune response against viruses, including SARS-CoV-2 [73]. IFIT2 (ISG54) is an anti-viral protein that inhibits the expression of viral mRNA and acts as a mediator of cell apoptosis [74]. Infection with the NSP1, NSP12, NSP13, NSP14, NSP15, NSP16, NSP2, NSP3, NSP6, ORF3a, ORF6, or ORF8 mutant resulted in 94, 3, 47, 35, 129, 18, 3, 60, 9, 19, 305, and 4 cellular genes, respectively, upregulated (log_2_FC ≥ 2 and *p*-value < 0.05) and 174, 15, 3, 1,122, 14, 15, 3, 1, 199, 799, 164, and 12 genes, respectively, downregulated (log_2_FC < 2 and *p*-value < 0.05) compared to that of WT SARS-CoV-2-infected cells (Fig 4C–4F).

Gene Ontology analysis of the upregulated genes in the Calu-3 cells infected with these mutants revealed a significant enrichment in immune and inflammatory processes with different degrees (Fig 4G). Additional analysis of the data for the NSP1 and NSP15 mutants—the two viruses, which demonstrated the greatest IFN-I response (Fig 2) and the greatest maturation of human DCs (Fig 3C) is shown in S4 Fig. Although NSP1, NSP3, NSP13, NSP15, and ORF6 mutants upregulated different sets of genes in Calu-3 cells, NSP1 and especially NSP15 mutants have uniquely activated genes that are related to the inflammatory processes and cytokine responses (Fig 4G). For example, for the NSP15 mutant, the upregulated pathways included cell response to cytokine signaling, defense response to virus, response to IFN-γ, cytokine-mediated signaling pathway, positive regulation of lymphocyte proliferation, and a few others (Fig 4G). Many of the genes upregulated by the NSP15 mutant are ISGs and are involved in innate defense against viruses. For example, the IFITMs are IFN-I-induced anti-viral proteins which inhibit SARS-CoV-2 replication [75], but sometimes promote it [76]. *IFNL1 to 3* are primarily expressed in epithelial cells with major anti-viral properties [34,77]. The NSP1 and NSP15 mutant viruses also activated many chemokine and pro-inflammatory cytokine genes that could play a protective role during infection. While pro-inflammatory genes (CCL22, CCL5, CX3CL1, CXCL9-11, IL-1B, MMP1, TNFSF13, and 15) facilitate inflammation, the immune regulatory genes (CIITA, SSA1, and CFB) could control excessive inflammation [78–80]. Importantly, IL-1β is important for adaptive immune cell differentiation and protective memory response [81,82].

**Fig 5:**
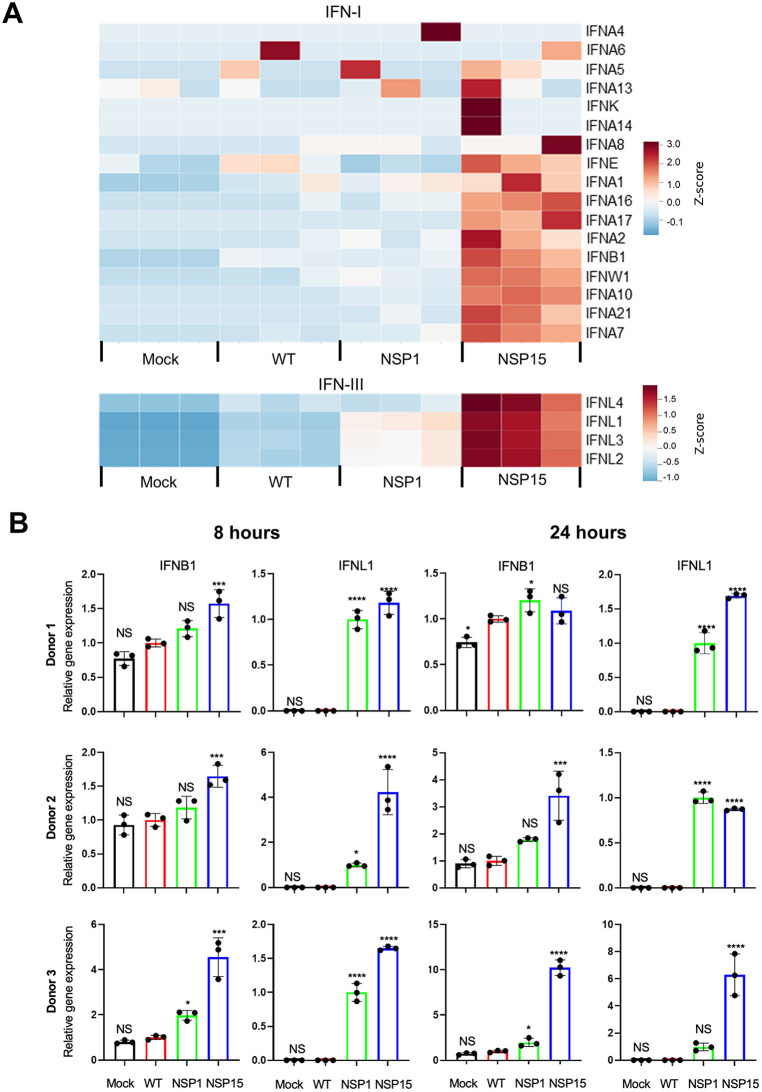
SARS-CoV-2 NSP1 and NSP15 suppress expression of IFN-I and IFN-III genes. **A.** Quantification of IFN-I and IFN-III by RNA-seq in Calu-3 cells infected with WT, NSP1 mutant, NSP15 mutant, or mock-infected. **B.** Quantification of IFNB1 and IFNL1 by qRT-PCR in human DCs infected with WT, NSP1 mutant, NSP15 mutant, or mock-infected. Statistical significance between each mutant and WT or NSP1 was assessed by one-way ANOVA, with significance levels indicated as follows: *p < 0.05, **p < 0.01, ***p < 0.001, ****p < 0.0001. NS, not significant. The data underlying panel B in this Figure can be found in S1 Data.

Further analysis of the NSP1 and NSP15 mutants demonstrated that infections with WT SARS-CoV-2 had a limited effect on the expression of most of IFN-I and IFN-III genes, while the NSP1 mutant also had a limited effect on the expression of IFN-I genes, but moderately increased expression of IFN-III genes (Fig 5A). In contrast, infection with the NSP15 mutant dramatically upregulated expression of many IFN-I genes and all IFN-III genes tested. Furthermore, the NSP1 and NSP15 mutations appeared to increase expression of IFN-I-inducible genes (S5A Fig) and reduce viral infection (S5B Fig), as compared to WT-infected cells. To determine the effect of NSP1 and NSP15 mutations on the expression of IFN-I and IFN-III in DCs, we infected monocyte-derived DCs from three human donors with WT SARS-CoV-2, NSP1, or NSP15 at an MOI of 3 PFU per cell or mock-infected, isolated RNA at 8 h and 24 h, and quantified IFNB1 and IFNL1 gene expression by RT-qPCR. Consistently, with the DC maturation data, the levels of mRNA for both IFN-I and IFN-III were low or nondetectable in WT SARS-CoV-2-infected cells, but were increased in cells infected with the NSP1 or NSP15 mutant (Fig 5B).

### SARS-CoV-2 NSP1 and NSP15 contribute to the viral pathogenicity in mice

**Fig 6:**
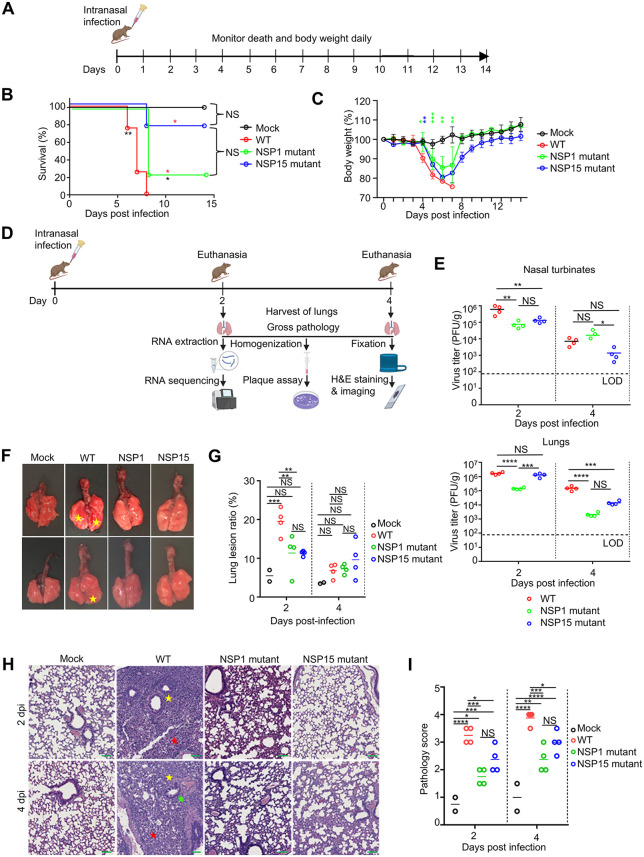
SARS-COV-2 NSP1 and NSP15 contribute the lethality and lung pathology. **A.** Experimental scheme of the mouse experiment 1 (panels A–C). Four groups of K18-hACE2 mice (*n* = 4 per group) were mock-infected or infected intranasally with WT SARS-CoV-2 or NSP1 or NSP15 mutant at 1 x 10^5^ PFU. Created in BioRender. Zhou, F. (2026) https://BioRender.com/s50n151. **B.** Survival. The red start(s) and black start are analysis results from the indicated virus compared to WT and mock respectively. **C.** Body weight change. **D.** Experimental scheme of the mouse experiment 2 (panels D–I). Eight groups of K18-hACE2 mice (two groups for each virus or mock infection, *n* = 4 per group) were mock-infected or infected intranasally with WT SARS-CoV-2 or NSP1 or NSP15 mutants at 10^5^ PFU, then euthanized at 2 or 4 days after the infection to harvest the lungs and nasal turbinates. **E.** Viral load in the nasal turbinates (top) and lungs (bottom). Created in BioRender. Zhou, F. (2026) https://BioRender.com/k01q783. **F.** Gross pathologic changes in the lungs. The lungs from WT virus-infected mice showed pneumonic changes of congestion and consolidation (above the asterisk mark), which were minimal or reduced in animals infected with the NSP1 or NSP15 mutant virus. **G.** Gross pathology scores in the lungs. **H.** Histology of typical interstitial pneumonic changes in the lungs, including severe inflammation with mononuclear cells and neutrophils (yellow asterisk), septal thickening (red asterisk), and perivascular changes (green asterisk) observed in lungs from WT virus-infected mice, while these changes were minimal or reduced in lungs from NSP1 or NSP15 mutant virus-infected mice. H&E staining. Scale bars (green), 100 µM. **I.** Histopathology score in the lungs. Data shown in panels E, G, and I represent mean ± SD from 4 animals. The significance was determined by log-rank (Mantel–Cox) test in panel B, A two-way ANOVA followed by Tukey’s multiple comparisons test in panel C, a one-way ANOVA test in panel E, and two-way ANOVA followed by Tukey’s multiple comparisons test in panels G, I. *p < 0.05, **p < 0.01, *** <0.001, ****p < 0.0001. The data underlying panels B, C, E, G, and I in this Figure can be found in S1 Data.

**Fig 7:**
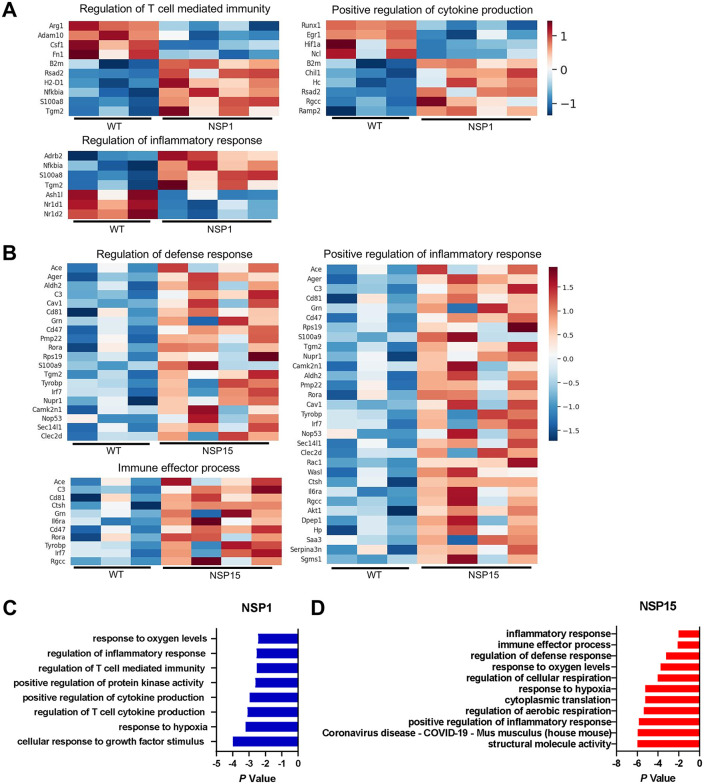
Bulk RNA-seq for transcriptome analysis of lungs in hACE2 transgenic mice infected by SARS-CoV-2 NSP1 and NSP15 mutants on day 4. Data from the mouse experiment 2 (Fig 5D). **A.** Changes in selected pathways for the NSP1 mutant vs. WT: regulation of T cell-mediated immunity, positive regulation of cytokine production, and regulation of inflammatory response. **B.** Changes in selected pathways for the NSP15 mutant vs. WT: regulation of defense response, immune effector processes, and positive regulation of the inflammatory response. **C.** Pathways upregulated for the NSP1 mutant vs. WT. **D.** Pathways upregulated for the NSP15 mutant vs. WT. The data underlying this Figure can be found in the GEO database, accession number GSE254969.

**Fig 8:**
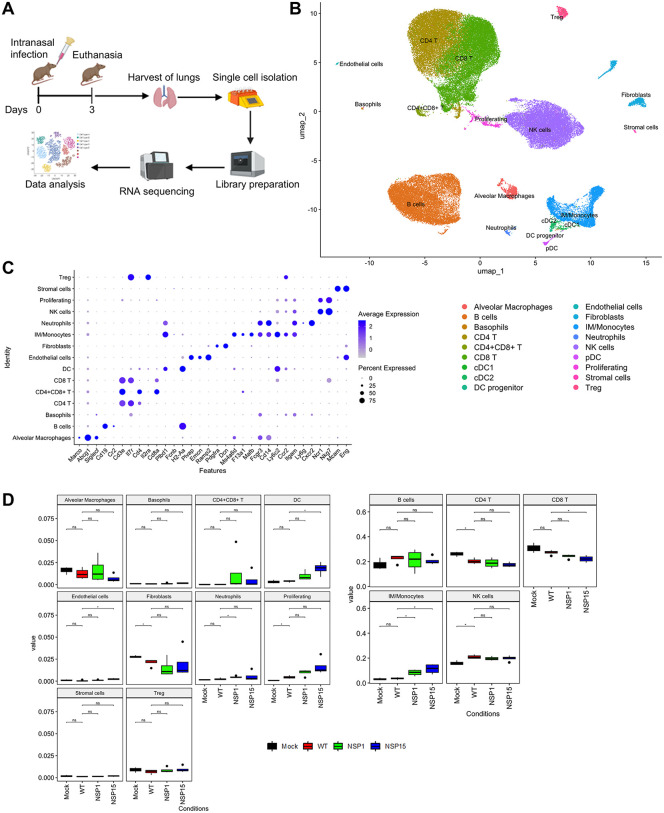
Single-cell RNA-seq analysis of pulmonary tissues identifies cell population differences between animals infected with WT, NSP1, or NSP15 mutant virus. **A.** Experimental scheme of the mouse experiment 3. Created in BioRender. Zhou, F. (2026) https://BioRender.com/i00o589. **B.** Integrated UMAP (uniform manifold approximation and projection) of all mock (*n* = 3), WT (*n* = 4), NSP1 (*n* = 4), and NSP15 (*n* = 4) samples. **C.** Dot plot showing canonical marker expression in observed lung cell types. **D.** Box plots comparing cell-type proportions observed in mock, WT, NSP1, and NSP15 groups. The limits of the box reflect the interquartile range (IQR: Q3–Q1) with the median shown as horizontal bars. Whiskers extend to 1.5 times the IQR of the box. For each cell type, pairwise *t* test comparisons of the wild type (WT) proportion with every other group are shown (* Holm *p*-adj < 0.1, ns - nonsignificant). The data underlying this Figure can be found in GEO database, accession number GSE255483.

We have shown that NSP1 and NSP15 suppress IFN-I signaling (Fig 2) and DC maturation (Fig 3). To investigate the impact of NSP1 and NSP15 on survival and gene expression in the context of in vivo infection, we used the K18 hACE2 transgenic mouse model of COVID-19 [83]. We performed three independent mouse infection experiments. In Experiment 1, we compared the clinical response of mouse (body weight change and lethality) following infection with WT SARS-CoV-2 or the NSP1 and NSP15 mutants, or PBS (mock infection) (Fig 6A–6C). In Experiment 2, infected mice were euthanized on days 2 and 4 post-infection for assessment of the viral load in nasal turbinates and lungs (Fig 6D, 6E), lung pathology (Fig 6F–6I), and transcriptome by bulk RNA-seq (Fig 7). In Experiment 3, infected mice were euthanized on day 3 to collect lung cells for scRNA seq analysis (Fig 8). In all the experiments, mice were infected intranasally with 10^5^ PFU of WT SARS-CoV-2 or NSP1 or NSP15 mutant. All the mice infected with WT SARS-CoV-2 succumbed to infection on days 6–8 (100% lethality), while the NSP1 and NSP15 mutants demonstrated 75% and 25% lethality, respectively (Fig 6B). Consistent with the lethality pattern, mice infected with WT SARS-CoV-2 lost 25% of their initial body weight, while mice infected with SARS-CoV-2 NSP1 and NSP15 mutants lost 15% and 17%, respectively, of their initial body weight (Fig 6C). Consistent with previous studies [84], WT SARS-CoV-2-infected mice demonstrated a very high viral load, both in the nasal turbinates and in the lungs (Fig 6E). NSP1 mutant virus-infected mice had significantly lower viral load in the nasal turbinates on day 2 and in the lungs on days 2 and 4 post-infection compared to WT virus-infected mice. NSP15 mutant virus-infected mice had significantly lower viral load in the nasal turbinates on day 2 and in the lungs on day 4 as compared to WT virus-infected mice (Fig 6E). The different levels of attenuation in the nasal turbinate versus the lungs could be related to differences in expression of IFN-I in these tissues. Sequencing of the mutated viral genome fragments in RNA isolated from mouse lungs on day 4 after infection in Experiment 2 demonstrated that all mutations were preserved.

Pathological examination of the lung tissues on day 2 showed areas of lung congestion and consolidation in mice infected with WT SARS-CoV-2. These changes were minimal in mice infected with the NSP1 and NSP15 mutants (Fig 6F, 6G). However, on day 4, the pathological changes in mice infected with NSP1 or NSP15 mutants were comparable to that in WT-infected mice (Fig 6F, 6G). On histological analysis, a typical interstitial pneumonia was noticed in lungs from WT SARS-CoV-2 infected mice on days 2 and 4 post-infection. The interstitial pneumonia was characterized by a severe inflammation with mononuclear cells and neutrophils in the alveolar space, interstitial septal thickening, and perivascular cuffing. In contrast, in mice infected with the NSP1 or NSP15 mutant, inflammatory changes were tempered with mild to moderate inflammation and minimal vascular and airway changes (Fig 6H). Histopathology scores of lung sections (Fig 6I and S2 Table) indicated that the NSP1 and NSP15 mutants caused less tissue damage than WT SARS-CoV-2 in the lungs. Altogether, these data indicate that SARS-CoV-2 NSP1 and NSP15 suppress the immune mechanisms which contribute to the protection against viral disease and death. The reduced tissue damage in the lungs of mice infected with the mutants, as compared to that infected with WT SARS-CoV-2, could be associated with the reduced viral load of the mutants, the biological effects of the mutated proteins, or both.

### SARS-CoV-2 NSP1 and NSP15 suppress innate immune responses in the lungs

As NSP1 and NSP15 contribute to viral pathogenicity in mice, we next investigated molecular pathways affected by the viral proteins in the lungs by comparison of transcriptome profiles in the lungs on day 4 by bulk RNA-seq analysis (mouse experiment 3). The DESeq2 analysis demonstrated 1,622 upregulated genes and 1,488 downregulated genes (log2 FC ≥ 2 and *p*-value < 0.01) in lungs of mice infected with WT SARS-CoV-2 compared to mock-infected mice. On comparison with mice infected with WT SARS-CoV-2, there were 61 and 22 upregulated genes [log2 fold change (FC) ≥2 and *p*-value < 0.01] and 44 and 25 downregulated genes (log_2_ FC ≥ −1.5, *p*-value < 0.01) in lungs of mice infected with SARS-CoV-2 NSP1 and NSP15 mutants, respectively (S6 Fig). Some of the genes upregulated by the two mutants are associated with effector immune functions, including Ace*, C3, Ctsh, Rora, Irf7, Rgcc, Saa3, Grn,* and *Dpep7.* Some of the downregulated genes, including *Arg1*, *Adam10*, *Robo2*, and *Smad2* are related to negative regulation of immune responses, suggesting that mutant viruses have activated the genes involved in effector immune functions. The heatmaps of DEG showed differences in patterns of gene expression between the NSP1 and NSP15 mutants on one hand and WT virus on the other hand (Figs 7A, 7B, and S6). In NSP1 mutant-infected mice upregulated genes included those involved in regulation of T cell immunity (B2m, h2d1, nfkbia, s100a8, and tgm2) and regulation of cytokines/inflammatory response (*b2m*, *nfkbia*, and *ramp2*) [85,86]. NSP15 mutant-infected mice, up-regulated genes included those involved in immune effector and inflammatory processes, particularly of innate immunity—*C3, CD81, Cd47, Ctsh, Grn, Il6ra, Rora, Tyrobp, Irf7, Rgcc, Kamk2n1, Cav1, Dpep1*, *Hp*, and *Saa3* (Fig 7B). Products of *C3, Ctsh, Grn, Il6ra*, and *Rora* genes are critical for functions of innate immune cells [87,88]. CD81 is important for B-cell differentiation and proliferation [89]. CD47 is an integrin-associated protein important for the transmigration of leukocytes [90]. Upregulation of Cd47 is a host checkpoint response to pathogen recognition, whose blockade can lead to enhanced immune response to pathogens that trigger PRR signaling [91]. *Tyrobp, Kamk2n1, Cav1,* and *Dpep1* regulate several protein kinases involved in immune cell function, particularly SYK, ERK1/2, MAPK, and PI3K/Akt/mTOR. IRF7 plays a vital role in the transcriptional activation of virus-inducible cellular genes and particularly IFN-I genes [92]. The *Saa3* and *Hp* genes encode acute phase proteins with pro-inflammatory and immunostimulatory effects [93,94]. Products of many of these genes are involved in immune effector functions. Importantly, the NSP1 and NSP15 mutants also downregulated, as compared to WT, expression *Runx1, Arg1, Junb, Cox5a, Adam10, Hif1a,* and *Serbp1*, that are transcriptional repressors and are involved in excessive inflammatory responses through hematopoiesis.

Pathway enrichment analysis revealed activation of genes in several pathways that are related to immune responses and other cellular functions for both the NSP1 and NSP15 mutants (Fig 7C, 7D). However, the changes in the pathway profiles induced by the two mutants were different. Together, bulk RNA-seq analysis revealed that NSP1 and NSP15 are involved in suppression and perturbation of immune effector functions that confer protective immunity against SARS-CoV-2.

### SARS-CoV-2 NSP1 and NSP15 suppress activation of immune functions in the lungs

To elucidate the effects of SARS-CoV-2 NSP1 and NSP15 on lung immune cells, we performed single-cell RNA-seq analysis of cells isolated from the lungs of K18 hACE2 transgenic mice on day 3 after infection with either WT SARS-CoV-2, the NSP1 or the NSP15 mutant, or after mock-infection (Fig 8A). We identified 15 major cell UMAP clusters: B cells, basophils, CD4 T cells, CD4+CD8+ T cells, CD8 T cells, DCs (the indicated subclusters are described below), endothelial cells, fibroblasts, alveolar macrophages, monocytes, neutrophils, NK cells, proliferating, stromal cells, and Tregs (Fig 8B, 8C). The alveolar macrophage population exhibited markers typical for these cells, including *Marco, Abcg1*, and *Siglecf* (Figs 8C and S7). The monocyte cluster was an intermixed population of cells that included both monocytes (marked by *Fcgr3, Cd14, Ly6c2*, and *Ccr2,* see Figs 8C and S7) and interstitial macrophages (marked by *Ms4a6d, F13a1, Mafb,* see Fig 8C). This co-clustering is consistent with the close relationship of interstitial macrophages and monocytes [95,96]. We refer to this cluster as interstitial macrophage (IM)/monocytes.

**Fig 9:**
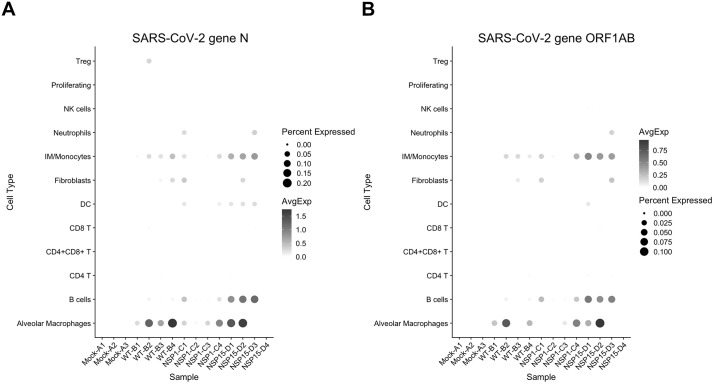
Viral reads in individual populations of immune cells isolated from mice infected with WT or mutated SARS-CoV-2. Dot plots showing the average expression of SARS-CoV-2 viral gene N (**A**) and ORF1AB (**B**) in each cell type/sample pair. Cell types with fewer than 20 cells in each sample are excluded from the plot. The value is not displayed in the plot (i.e., set to zero) for any cell type/sample pair that has fewer than 20 cells. The data underlying this Figure can be found in GEO database, accession number GSE255483.

In comparison with mock infection, WT infection resulted in a significant increase in the proportion of NK and proliferating cells and a significant reduction in the proportion of CD4+ T cells and fibroblasts. We also observed a reduction in the proportion of alveolar macrophages and CD8+ T cells that did not reach significance. These changes support immunosuppression by the WT virus. Infection with the NSP1 and NSP15 mutants resulted in an increase in the proportion of interstitial macrophages/monocytes (IM/monocytes) as compared to WT-infected mice. Moreover, the relative expression levels of some monocyte and IM markers were differentially affected by WT and mutant infections (S8 Fig). For example, the microenvironment modulatory IM marker C5ar1 is dramatically increased only after infection with the NSP15 mutant (S8 Fig). These results indicate that NSP1 and NSP15 have specific effects on the state of IM/monocytes. The proportion of double-positive CD4+/CD8+ T cells was increased with the mutant infections as compared to mock-infected and WT virus-infected mice. In addition, infection with the NSP15 mutant also resulted in an increase in the proportion of DCs and a reduction of CD8+ T cells; similar trends were observed in infection with the NSP1 mutant without reaching statistical significance (Fig 8D). Analysis of the viral transcripts demonstrated viral reads (N or ORF1AB) of WT and NSP15 mutant viruses, and very low levels of the NSP1 mutant virus. Viral RNA was detected alveolar macrophages, that is consistent with published data [97], monocytes, and also in B cells, but the levels of expression were inconsistent across the groups (Fig 9). A somewhat greater abundance of the NSP15 mutant viral reads, as compared to WT viral reads, in B cells and AMs could be related to a slower kinetics of the NSP15 mutant replication in the lungs. Overall, these data suggest that NSP1 and especially NSP15 contribute to the immunosuppression and dysregulation of the immune response caused by SARS-CoV-2.

**Fig 10:**
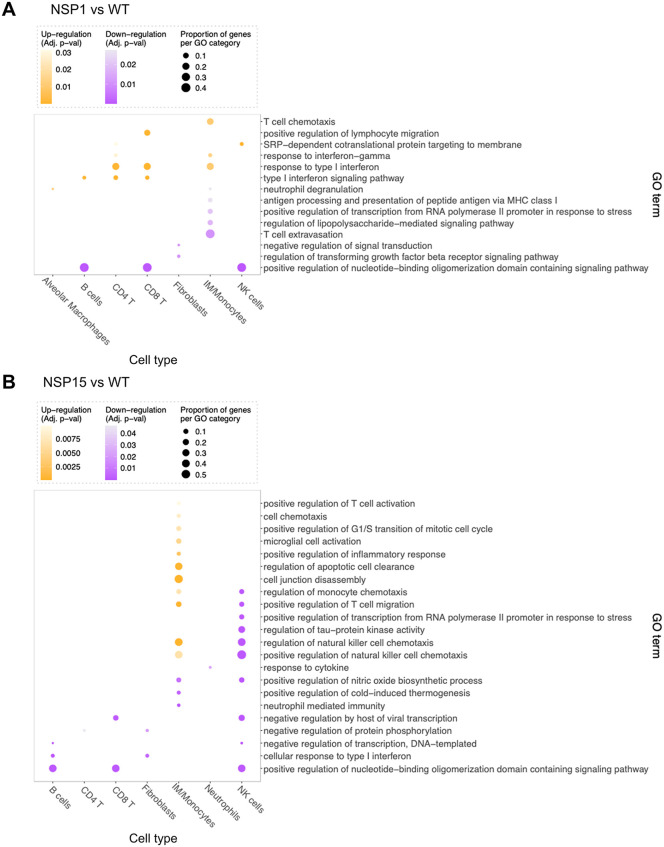
Changes in activation of pathways associated with the NSP1 and NSP15 mutants. Dot plots showing GO terms from pathway enrichment analyses by EnrichR among differentially expressed genes (DEG) for the NSP1 vs. WT **(A)**, NSP15 vs. WT **(B)**. Dot size represents the fraction of DEG within the GO term. Dot color represents the direction of the regulation of the term in the corresponding cell type (up-regulation: yellow; down-regulation: purple) and the color scale indicates the adjusted *p*-value (shown are only terms with FDR adjusted enrichment *p*-value < 0.1). Terms with substantial gene overlap are filtered out, with terms remaining only if there is a difference of at least two regulated genes from every other term. The direction of regulation for each enriched term is determined by the proportion of upregulated DEG vs. the downregulated DEG across all cell types. The data underlying this Figure can be found in GEO database, accession number GSE255483.

Differential gene expression analysis of WT virus-infected mice compared to mock showed substantial regulatory remodeling of multiple cell types (S9A Fig). In particular, there were 242 upregulated and 109 downregulated genes in IM/monocytes. Additionally, in all cell types analyzed other than proliferating cells, WT virus infection resulted in a regulatory program that included many more upregulated genes compared to downregulated genes (S9A Fig, S3 Table). Pathway analysis revealed enrichment of several pathways linked to immune effector functions, primarily the innate immunity and the cellular metabolic pathways in response to WT virus infection, in all analyzed cell populations, with a somewhat lesser number of activated pathways detected in DCs and neutrophils (S10 Fig). Alterations in the regulatory program between the mutants and WT virus were also observed (S9B, S9C Fig). In comparison to WT virus, the NSP1 mutant showed upregulation of IFN-I response in IM/monocytes and T cell populations (Fig 10A). In addition, the mutant showed downregulation of nucleoside-binding oligomerization domain (NOD) genes for some lymphoid cell populations. The NSP15 mutant, on the other hand, activated multiple pathways in IM/monocytes, but down-regulated several pathways in NK cells (Fig 10B). Together, these data suggest that both NSP1 and NSP15 mutants suppress multiple pathways associated with antiviral defense, but the effects of these two proteins are different.

**Fig 11:**
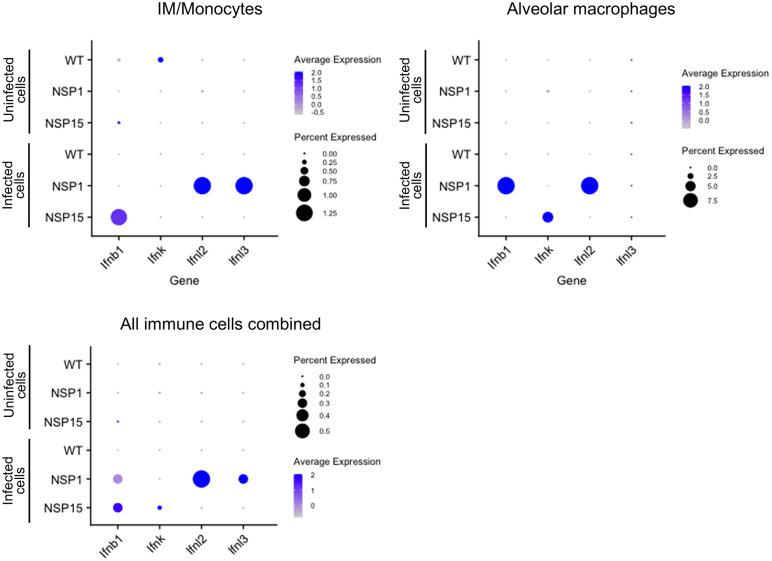
Expression of the indicated IFN-I genes in intersticial macrophages (IM)/monocytes, alveolar monocytes, and all immune cells combined in uninfected and infected cells assessed by single-cell sequencing. Color indicates average expression values and size indicates the percentage of cells expressing the transcript. The data underlying this Figure can be found in GEO database, accession number GSE255483.

The number of cells with detectable viral RNA was relatively low, limiting expression analysis to only the most abundant genes. Analysis of the responses in virus transcript-expressing and nonexpressing cells demonstrated greatly increased expression of IFNß, IFN-λ2, IFN-λ3 in mice infected with NSP1 and IFNβ and IFN-κ in IM/monocytes, alveolar macrophages and all cell types aggregated together isolated from mice infected with NSP15 compared to WT-infected mice (Fig 11).

To compare the activation of pulmonary DCs, we analyzed DC maturation markers CD80 and CD86. The number of DCs captured was too small to allow differential analysis. The expression of CD80 and CD86 was higher in samples from the NSP1 and NSP15 mutant-infected mice as compared to WT virus-infected mice (S11A Fig). Moreover, DCs from the NSP1 and NSP15 mutant infected mice demonstrated greater expression of IFN-I-inducible genes ISG15 and OAS1G (S11B Fig). To perform a more detailed analysis of DCs, we subclustered the DC population identified in the UMAP shown in Fig 8B, which enabled us to identify 7 clusters comprising 5 DC subsets: cDC1, cDC2, DC progenitors, pDC, and pDC with low expression of CCR9 (S12A, S12B Fig) [98]. The proportions of the DC subsets tended to be greater in the NSP1 and NSP15 mutant-infected mice as compared to WT virus-infected mice, although in many cases the difference did not reach statistical significance (S12C Fig).

Overall, the single-cell sequencing data demonstrate the suppression of the activation of immune cells in the mouse lung by NSP1 and NSP15 further supporting the conclusions of the data with human DCs (Figs 3 and 5B) and are consistent with the data on 293T-ACE2/TMPRSS2 cells (Fig 2), Calu-3 cells (Figs 4 and S4, S5), and bulk sequencing of the mouse lungs (Figs 7 and S6).

## Discussion

Here, we focused on side-by-side comparison of the effects of multiple SARS-CoV-2 proteins which antagonize the innate immune response. We selected multiple genes of SARS-CoV-2 based on their significance for viral pathogenicity and inhibition of the host immune responses (Fig 1A, Table 1) to determine: (1) their contribution to immunosuppression in the context of viral infection, rather than a plasmid-based system, and (2) to compare their relative contribution to immunosuppression relative to each other side-by-side in multiple experimental systems. The studies showed that all 13 genes play a role in IFN-I antagonism (Table 1). Based on the information, we generated 16 full-length cDNA BAC clones with mutations in the 13 different genes. Among them, 12 clones resulted in a successful recovery of infectious viruses, while no virus could be recovered from the other four clones (Table 1).

We used the ISRE-driven luciferase assay in 293T-ACE2/TMPRSS2 cells to first test the 12 mutated viruses with and without IFN-α treatment. Remarkably, seven of them demonstrated induction of IFN-I response in at least one of the two systems (Fig 2). In IFN-α-treated cells, the NSP1 and NSP15 mutant viruses demonstrated the greatest response. NSP1 interacts with the human 40S to interfere with mRNA binding and translation. SARS-CoV-2 NSP1 with the mutations K164A/H165A lost its ribosome binding and inhibition capability [28]. NSP15 is a uridine-specific endoribonuclease conserved across the *Coronaviridae* family [99] and mediates the evasion of host recognition of viral dsRNA [100]. Based on the comparison of SARS-CoV-2 NSP15 with those of the other 11 coronaviruses, including SARS-CoV and MERS-CoV [100], we speculate that the residue H234 is involved in endoribonuclease activity. Interestingly, the NSP1 but not the NSP15 mutant formed smaller plaques compared to WT SARS-CoV-2 (Fig 1B), suggesting different effects of the proteins on viral replication.

In COVID-19 patients, SARS-CoV-2 inhibits IFN-I response and reduces the number of DCs, and both effects contribute to the progression of severe COVID-19 [101]. Additionally, in COVID-19 convalescent patients, the DC maturation markers are suppressed for months after the onset of the disease [66,102]. Strikingly, the NSP1 and NSP15 mutants, which demonstrated the greatest IFN-I signaling (Fig 2), induced the greatest DC maturation (Fig 3A–3C). IFN-I is known to enhance DC maturation in combination with other stimuli, such as ligation of toll-like receptors and costimulatory molecules by viral proteins [103], and IFN-I is therefore an important link between innate and adaptive immunity through DC stimulation [104]. These effects are consistent with the role of IFN-I in COVID-19 patients, in which a highly impaired IFN-I response was associated with a persistent blood viral load and an exacerbated inflammatory response [10].

Based on the screening of the mutants for their ability to suppress IFN-I response and maturation of DCs, we selected the NSP1 and NSP15 mutants for in-depth characterization in vitro and in vivo. Infection of Calu-3 cells and human DCs with the NSP1 and NSP15 mutants activated IFN-I and IFN-III genes, and a set of genes that are linked to immune functions and inflammatory processes, demonstrated in Calu-3 cells (Figs 4, S4, and S5A), indicating that SARS-CoV-2 interferes with protective immune responses through these proteins. Notably, many genes upregulated by the NSP1 and NSP15 mutant viruses are responsive to IFN-I stimulation (ISG) and are involved in innate defense against viruses, underlying the role of NSP1 and NSP15 in IFN-I antagonism.

Evaluation of the two selected SARS-CoV-2 mutants NSP1 and NSP15 in K18 hACE-2 transgenic mice demonstrated the reduced viral load, inflammation, and pathogenicity as compared to the WT virus (Fig 6). Analysis of the lung transcriptome from the NSP1 mutant-infected mice demonstrated marked changes in pathways associated with regulation of T cell-mediated immunity, inflammatory response, and cytokine production (Fig 7). The NSP15 mutant gave a more straightforward change: upregulation of defense response, immune effector processes, and inflammatory response. These data clearly suggest that NSP1 and NSP15 mutant viruses induced mild to moderate inflammation, as opposed to the severe inflammation noted in mice infected with WT virus. Mice infected with NSP1 and NSP15 mutants had fewer inflammatory cells in the lungs on histological analysis when compared to the WT virus. The monocytes in the lungs of mice infected with the mutant viruses, especially the NSP15 mutant, had upregulation of several genes and pathways related to immune effector functions (Figs 8, 10, and 11), compared to the WT virus-infected animals. Overall, these data indicate that NSP1 and NSP15 inhibit the IFN-I pathways and activation of effector immune cells, contributing to severe disease.

The bulk RNA-seq (Fig 7) and scRNA-seq (Figs 8, 10, and 11) of the lung tissue demonstrated that the NSP1 and NSP15 mutant viruses differentially activated the genes related to the inflammatory process, T cell-mediated immunity, immune effector functions, and positive regulation of cytokine production. scRNA-seq revealed a higher proportion of monocytes and DCs during NSP1 and NSP15 mutant infections, which were associated with reduced lethality (Fig 6). Importantly, the proportion of inflammatory monocytes was higher in the lungs of mice infected with the NSP1 and NSP15 mutant viruses when compared to those mice infected with WT virus (Fig 8D), suggesting a protective role of these cells. Multiple gene pathways related to the protective innate immunity, positive regulation of inflammatory response, IFN-I signaling, T cell-mediated immunity, and immune effector functions were activated in mice infected with the NSP1 and NSP15 mutant viruses (Figs 7, 8, 10, and 11). The effects of NSP1 or NSP15 mutations on these responses suggest specific immunosuppressive mechanisms of the two viral proteins. Interestingly, scRNA-seq analysis identified downregulation of NOD pathways in B cells, CD8+ T cells, and NK cells after infection with the NSP1 and NSP15 mutants (Fig 10). This suggests that NSP1 and NSP15 exhibit control over inflammasome activation.

When this work was in progress, two important studies were published. Stauft and colleagues reported the use of attenuated SRS-CoV-2 with mutations in NS1 identical to those used by us [105]. Similarly to our study, the virus was highly attenuated in vivo. Respiratory tract immunization of hamsters with the virus induced mucosal and systemic IgA and IgG and effectively protected animals from multiple strains of SARS-CoV-2. Otter and colleagues used mutations in NSP15 identical to those used by us [31]. The authors demonstrated that the mutated virus induced increased activation of IFN-I signaling and PKR pathways in cultured cells and attenuation in mice, that is consistent with our data. The novelty of the corresponding components of our study, as compared to the two cited studies, includes the investigation of the effects of the mutated domains on the maturation of human DCs and on activation of multiple populations of immune cells in mouse lungs.

The study has limitations. It did not result in the assessment of the contribution of NSP5 and NSP6 in the viral immunosuppression, as the viruses with the mutations in these genes were not recoverable. Furthermore, immunosuppressive effects of the nonmutated parts of the selected proteins, as well as of additional SARS-CoV-proteins, which were not included in the study due to the lack of information on their possible immunosuppressive effect when the project was conceived, cannot be excluded. While this study addresses downstream effects of IFN-I antagonism of SARS-CoV-2 proteins, focusing on immunopathogenesis, it does not address the mechanisms by which these proteins antagonize IFN-I response. We note that other studies address these mechanisms for NSP1 [14,17,106] and NSP15 [23,31]. Finally, inflammatory mechanisms involving IL-1 and IL-1ra are different in human and mouse cells [107].

In conclusion, this work demonstrates both distinct and redundant mechanisms of immunosuppression of SARS-CoV-2 mediated by multiple individual viral proteins, with 9 out of the 12 tested proteins showing some immunosuppressive effect in at least one experimental system (S13 Fig). Notably, our findings reveal that immune antagonism by SARS-CoV-2 is not dominated by a single factor but instead arises from coordinated, partially redundant activities of multiple proteins that converge on shared immune pathways. The demonstrated immunosuppressive effects extend from the innate response to immune cells to pathologic changes in vivo. Beyond establishing comparative effects, our data demonstrate that individual viral proteins differentially couple IFN-I and IFN-III antagonism to downstream suppression of DC maturation, immune cell activation, and in vivo pathogenicity, with NSP1 and NSP15 emerging as dominant and mechanistically distinct regulators. These findings provide an integrated view linking specific viral immune evasion strategies to functional outcomes across cellular and organismal levels, which has not been resolved in prior studies. Importantly, this work shows, for the first time, a comparison of the effects of multiple viral proteins in the context of authentic viral infection, rather than in a surrogate system, and shows the relative contribution of each viral protein under identical experimental conditions, as opposed to different experimental systems in individual studies.

## Methods

### Ethics statement

The mouse experiments were approved by the University of Texas Medical Branch (UTMB) Institutional Care and Use Committee (IACUC) (protocol 2204022), Animal Care and Use Review Office of the U.S. Department of the Army (under UTMB IACUC protocol number 2204022), and the Texas Biomedical Research Institute IACUC (protocol 1718MU).

### Sex as a biological variable

Our study exclusively examined female mice. There are no indications of any significant difference between male and female mice in response to SARS-CoV-2. The selection of female mice was also related to limited space in BSL-3 containment.

### Generation of mutated SARS-CoV-2 constructs

The full-length SARS-CoV-2 strain USA-WA1/2020 cDNA clone pBeloBAC11-SARS-CoV-2 [108] was transformed into *E.coli* strain GS1783 (kindly provided by Dr. Gregory A. Smith), and a lambda red system-based En Passant mutagenesis was adapted to introduce point mutations or deletions. The Red system, which originates from λ phages, allows for the insertion of linear double-stranded DNA molecules by homologous recombination, and it consists of three proteins named Exo, Beta, and Gam [109]. The method uses short identical DNA sequence-mediated recombination between linear DNA and circular DNA to insert DNA sequences into BAC DNA and to remove the marker cassette from the BAC DNA in a second Red recombination step in *e.coli* strain GS1783 with expression of Lambda Red recombination genes (Exo, Beta, and Gam) or/and the I-SceI enzyme under the control of temperature-inducible and arabinose-induced promoters, respectively [61]. The final constructs with the selected mutations were generated through the two rounds of recombination. In brief, fragments were amplified from pKan-S with primers, containing the desired mutations, the sequences homologous to I-SceI/Kan resistance cassette, and the gene intended to be mutated. The primers used to amplify the I-SceI/Kan^r^ cassette to introduce mutations are shown in S1 Table. The first recombination between the PCR fragment and pBeloBAC11-SARS-CoV-2 resulted in insertion of the I-SceI/Kan^r^ cassette under kanamycin selection. The cassette carries an I-SceI site and is flanked by two identical short sequences with the desired mutation. The second round of recombination between the two identical sequences removes the I-SceI/Kan^r^ cassette, resulting in the construct with the desired mutations only. To confirm the mutations, the mutated region was PCR-amplified from the mutated constructs for DNA sequencing. To further validate the mutated full-length SARS-CoV-2, DNA was characterized, in parallel with the parental nonmutated clone, by BsmBI restriction endonuclease.

### Recovery of SARS-CoV-2 mutants

The full-length clone pBeloBAC11-SARS-CoV-2 and its mutated derivatives were used for recovery of SARS-CoV-2 mutants using the previously described method [108] with some modifications. The procedure was performed under BSL-3 containment of the Galveston National Laboratory. Briefly, 80% confluent monolayers of Vero E6 cells in 12-well plates were transfected with 1.0 μg per well of infectious SARS-CoV-2 BAC DNA or its mutated derivatives using TransIT-LT1 transfection reagent according to the manufacturer’s instructions (Mirus Bio). At 48 h post-transfection, the transfected cells were split and seeded into 25 cm^2^ flasks with 50% confluent monolayers of Vero E6 cells. The cell cultures were observed daily until the appearance of a strong cytopathic effect to collect viral supernatants. To confirm the desired mutation in the recombinant viral mutants, DNA-free viral RNA was isolated from the viral supernatants using Direct-Zol RNA Microprep kit (Zymo research) according to the manufacturer’s instruction. The expected sequences of the viral genomes were further confirmed by reverse transcription-PCR followed by Sanger DNA sequencing.

### Virus titration

Vero E6 cells were seeded on 96-well plates or 12-well plates. On the next day, the cells were incubated with 10-fold serial dilutions of the virus for 1 h and overlaid with 0.55% methylcellulose or 1.0% cellulose in minimal essential medium (MEM) (Gibco) supplemented with 2% FBS (Corning) and 50 µg/ml gentamycin (Corning/Fisher). If methylcellulose was used, the overlay was discarded, the infected cells were fixed with 10% formalin for 24 h, then stained with 10% formalin containing 0.25% crystal violet at room temperature for 15 min and washed with water or stained with a mixture of 37 human monoclonal antibodies targeting different antigenic sites within spike protein of SARS-CoV-2 [110]. Then the plaques were visualized using HRP-conjugated anti-human IgG with Nova-red substrate kit (Vector Laboratories). If cellulose was used, the overlay was discarded, and the infected cells were fixed with 10% formalin for 24 h, and the monolayers were stained with 10% formalin containing 0.25% crystal violet at room temperature for 15 min and washed with water.

### Growth kinesics

To assess replication kinetics of the panel of 13 viruses, duplicate Calu-3 cell monolayers in 12-well plates were infected with the indicated viruses at the MOI of 0.01 PFU per cell. Following a 1 h adsorption at 37 °C, the inoculum was removed and the cells were washed three times with PBS. Subsequently, 1 mL of DMEM supplemented with 2% FBS and 50 µg/mL gentamycin was added to each well. At the indicated time points post-infection, 100 µL of supernatant was collected from each well and stored at −80 °C. After each collection, the sampled volume was replaced with 100 µL of fresh medium to maintain a constant volume of 1 mL per well. Virus in supernatants was titrated by plaque assay in 96-well plates as described above. To assess replication kinetics WT virus, NSP1, and NSP15 mutant viruses in Vero-AT cells (NR-54970, BEI Resources) and A549-hACE2 cells (NR-53821, BEI Resources), triplicate monolayers in 6-well plates were infected at an MOI of 0.01 PFU per cell. After 1 h adsorption at 37 °C, the inoculum was removed, and the cells were washed three times with PBS. Each well was then supplemented with 3 mL of DMEM containing 2% FBS and 1% penicillin-streptomycin-glutamine (PSG). At designated time points post-infection, 300 µL of supernatant was collected, aliquoted, and stored at −80 °C. Viral titers were subsequently determined by plaque assay. For plaque visualization, confluent duplicate monolayers of Vero-AT or A549-hACE2 cells were seeded in 6-well plates at 1 × 10⁶ cells per well and infected with 10-fold serial dilutions of virus for 1 h at 37 °C. After adsorption, the inoculum was removed, and cells were overlaid with post-infection medium containing 1% low-melting agar and incubated at 37 °C for 60 h. Cells were then fixed overnight with 10% formalin at 4 °C. For immunostaining, cells were permeabilized with 0.5% Triton X-100 in PBS for 15 min at room temperature and stained using the monoclonal antibody 1C7C7 specific for the SARS-CoV-2 N protein (1 µg/mL) in combination with the Vectastain ABC kit, following the manufacturer’s instructions. Plates were imaged using a flatbed scanner (Epson).

### ISRE-luciferase reporter assay

293T-ACE2/TMPRSS2 cells, which constitutively express SARS-CoV-2 receptors ACE-2 and TMPRSS2 (ACE2/TMPRSS2) [64] were plated onto poly-L-lysin-coated 96-well plates (Corning, cat. #3917), and one day later the cells were transfected with pISRE-luc and pTK-RL (Promega) using TransIT-LT1 Transfection Reagent. pISRE-luc has a firefly luciferase gene driven by a minimal promoter with IFN-stimulated responsive element (ISRE) and pTK-R encodes a constitutively expressed *Renilla* luciferase as a transfection control. Twenty-four h later, the cells were mock-infected or infected with SARS-CoV-2 or its mutants at 0.3 PFU/cell. Twenty-four hours after the infection, the cells were untreated or treated with universal IFN (PBL Assay Science) at 100 uints/ml. Twenty-four hours after the treatment, the reporter assay was performed using Dual-Glo Luciferase System (Promega, Cat# E2920) according to the manufacturer’s instructions. The luminescence in the wells of the 96-well plates was measured using the Cytation 7 reader (BioTek Instruments).

### Immunofluorescence assay

To compare the percentages of cells infected by the viruses, 293T-ACE2/TMPRSS2 cell monolayers on coverslips in 24-well plates were infected at the MOI of 0.3 PFU per cell with the panel of viruses in triplicate and incubated the monolayers for 24 h. The cells were fixed with 4% paraformaldehyde for 15 min at room temperature, washed with PBS, and permeabilized and blocked with PBS containing 0.5% saponin and 5% normal goat serum for 45 min. After wash, the cells were incubated with rabbit anti-SARS NSP3 antibody (ab181620, Abcam), which can recognize SARS-CoV-2 NSP3 [111] for 1 h followed by goat anti-rabbit IgG (H + L) cross-adsorbed secondary antibody labelled with Alexa Fluor 594 (A-11012, Thermo Scientific) for 1 h. Nuclei were stained with DAPI (Sigma-Aldrich). The images were collected with an Olympus IX73 fluorescence inverted microscope.

### Human dendritic cell maturation assay

Human peripheral blood mononuclear cells (PBMC) were used for the isolation of primary monocytes. The PBMC were collected by gradient centrifugation using Histopaque-1077 (Sigma) from the commercially sourced buffy coats (Gulf Coast Regional Blood Center, Houston, Texas, USA) of three unidentified healthy donors. The monocytes were isolated from PBMCs by magnetic positive selection with MidiMacs separation columns (Miltenyi Biotech) and anti-CD14 antibody-coated magnetic beads (Miltenyi Biotech). Purified monocytes were cultured in monocyte-derived DC differentiation medium containing IL-4 and GM-CSF (Miltenyi Biotech) for 6 days at 37 °C in humidified air containing 5% CO_2_. Cells were replenished with fresh medium on day 3. The procedure resulted in differentiation of monocytes into monocyte-derived DCs with an immature phenotype (CD11c^+^/CD14^−^). On day 6, DCs were harvested using enzyme-free PBS-based cell dissociation buffer (Gibco) and washed in RPMI-1640 medium (Gibco) supplemented with 10% FCS, 1% penicillin/streptomycin, and 1% pyruvate. Cells were counted and aliquoted in duplicates in 5 ml Falcon tubes (5 x 10^4^ cells in 100 µl medium per tube). Cells were treated with LPS (1 μg/ml) or SARS-CoV-2 (MOI 3 PFU/cell) under BSL-3 containment and cultured for 1, 2, or 3 days. After incubation, DC maturation status was analyzed by multicolor flow cytometry using anti-human antibodies specific for CD11c (clone BU15), CD80 (clone W17149D), CD86 (clone BU63), and MHCII/HLA-DR (clone LN3) (Biolegend). Cells were incubated with a cocktail of antibodies for 10 min at room temperature, washed once with PBS, fixed twice with 4% PFA and removed from BSL3 containment. The fixed cells were analyzed using the LSRFortessa flow cytometer (BD Biosciences). After exclusion of cell debris from the analysis by forward and side scatter gating, the cells were gated for CD11c+ population, which was further gated for the expression of CD80, CD86, or HLA-DR. The percentages of cells expressing the maturation markers and their MFI values were calculated in FlowJo software v10.8 (BD).

### Quantification of IFNβ and IFN-λ by RT-qPCR

Human monocyte-derived DCs from three human donors were mock-infected or infected in triplicates with WT SARS-CoV-2, or NSP1 and NSP15 mutants. At 8 and 24 h post-infection, cells were lysed in TRIzol, and total RNA was isolated using the Zymo microRNA extraction kit. cDNA was generated from the purified RNA using the High-Capacity cDNA Reverse Transcription Kit. Quantitative PCR was performed using TaqMan Universal PCR Master Mix together with gene-specific assays for IFNA1 (Hs00256882_s1), IFNB1 (Hs00277188_s1), IFNL1 (Hs00601677_g1), and GAPDH (Hs02758991_g1) (Applied Biosystems). Relative gene expression was calculated using the comparative Ct (ΔΔCt) method. Ct values were normalized to GAPDH to obtain ΔCt values, and ΔΔCt values were calculated relative to mock-infected controls, or to NSP1 mutant-infected samples when expression was undetectable in wild-type-infected cells. Fold changes in gene expression were expressed as 2^−ΔΔCt relative to wild-type or NSP1-infected conditions.

### Isolation of RNA from infected Calu-3 cells or homogenized mouse lungs

The infected cells or homogenized lungs were lysed in Trizol to prepare total RNAs using Direct-Zol RNA Microprep kit (Zymo Research) with DNase treatment according to the manufacturer’s instructions. The isolated RNA was submitted for RNA sequencing.

### Preparation of cDNA library and analysis of the RNA-seq data

For Calu-3 cell-derived RNA, the concentration and integrity (RIN) of isolated RNA were determined using the Quant‐iT RiboGreen RNA Assay Kit (Thermo Fisher) and an RNA Standard Sensitivity Kit (Agilent Technologies, catalog number DNF‐471) on a Fragment Analyzer Automated CE system (Agilent Technologies). Subsequently, cDNA libraries were constructed from total RNA using the Universal Plus mRNA‐Seq kit (Tecan Genomics) in a Biomek i7 Automated Workstation (Beckman Coulter). Briefly, mRNA was isolated from purified 300 ng total RNA using oligo‐dT beads and used to synthesize cDNA following the manufacturer’s instructions. The transcripts for ribosomal RNA (rRNA) and globin were further depleted using the AnyDeplete kit (Tecan Genomics) prior to the amplification of libraries. Library concentration was assessed fluorometrically using the Qubit dsDNA HS Kit (Thermo Fisher), and quality was assessed with the HS NGS Fragment Kit (1–6,000 bp; DNF‐474, Agilent Technologies).

Following library preparation, samples were pooled and preliminary sequencing of cDNA libraries (average read depth of 90,000 reads) was performed using a MiSeq system (Illumina) to confirm library quality and concentration. Deep sequencing was subsequently performed using an S4 flow cell in a NovaSeq sequencing system (Illumina; average read depth ~30 million pairs of 2 × 100 bp reads) at the New York Genome Center. Reads were subjected to quality control using FastQC (v0.11.8) and RNASeqMetrics (Picard v2.18.16), mapped using STAR (v2.7.0d), and gene counts were summarized using RSEM (v1.3.1). Counts were normalized using DESeq2 (v1.30.1) regularized-log transform function, following a differential gene expression analysis. GO enrichment analyses were performed using the R packages Go.db (v.3.7.0) and GOstats (2.48.0).

Lung-derived mRNA sequencing was conducted according to the SMART-3SEQ method [112]. Concisely, the first strand primer was annealed to 1 µl of sample RNA before being extended with SMARTScribe reverse transcriptase (Clontech). After the addition of the second strand primer, second strand synthesis was carried out, and adapter sequences with unique indexes were inserted with 15 cycles of PCR using NEB Next single index adapters (New England BioLabs). Purified PCR products were purified using AMPure XP SPRI beads (Beckman Coulter Life Sciences) and pooled for sequencing on an Illumina NextSeq 550 High Output Flow Cell using the single-end 75 base technique using AMPure XP SPRI beads (Beckman Coulter Life Sciences).

A five-base unique molecular identification (UMI) and 3Gs were added to the 5′ end of every sequence using the Smart-3SEQ protocol. Using STAR version 2.7.5c and the settings suggested by the software developers for the Encode consortium, reads were aligned to the Mus musculus NCBI assembly GCF_000349665.1. The NCBI annotation version 102 was utilized to count reads per gene using the FeatureCounts software. The count table was utilized as an input into DESeq2, allowed for the estimation of differential gene expression in accordance with the DESeq2 vignette that was provided with the software. The R heatmap tool was used to do hierarchical clustering of the genes. Furthermore, Metascape was used to perform pathway enrichment analysis on the set of differentially expressed genes with *p* values < 0.05. The enriched pathways were identified using the Gene Ontology terms biological process, molecular function, and KEGG pathway. Pathways were chosen with an FDR adjusted *p* value < 0.01. All heatmaps were created using the Python 3.9 libraries matplotlib and Seaborn. To normalize the ISG expression for virus-infected samples, we computed a normalization factor based on the average of viral gene expression over eight selected viral genes (S, ORF1ab, ORF3a, ORF7a, ORF7b, ORF8, ORF10, and M). This normalization factor was the ratio of the average viral gene expression in each infection condition to the maximum average viral gene expression (S5A, S5B Fig). For viral gene expression (Fig 5B), median-based normalization was performed using DESeq2. Viral gene expression was computed by mapping the original fastq files against an index generated from the SARS-CoV-2 genome. All further quantification was done using the standard RSEM pipeline.

### Infection of K18 hACE2 transgenic mice

The mouse experiments were conducted in an animal biosafety level 3 (ABSL-3) facility of the Galveston National Laboratory and the Texas Biomedical Research Institute. The K18 hACE2 transgenic mice B6.Cg-Tg(K18-ACE2)2Prlmn/J were purchased from the Jackson Laboratory. The following three experiments were conducted:

1. Infection of mice to assess the survival and disease. Six-week-old K18 hACE2 transgenic mice at 4 animals per group, a total of 16 mice, were sedated with isoflurane and either mock-infected with medium or infected intranasally with 10^5^ PFU of SARS-CoV-2 or the mutants with a final volume of 50 µl (~25 μl into each nostril). After infection, mice were monitored daily for survival and body weight. Mice showing >25% loss of their initial body weight were defined as reaching the experimental endpoint and humanely euthanized.
2. Infection of mice to assess viral load and change in gene expression in lungs. Six-week-old female K18 hACE2 transgenic mice at 8 animals per group, a total of 32 mice, were either mock-infected with medium or infected as described above. The mice were euthanized at 2 and 4 days post-infection, and the lungs were isolated for assessment of gross changes. The whole lungs were excised and photographed. Next, the left lungs were fixed in 10% neutral buffered formalin for histopathology, and the right lungs were processed for virus titration and RNA isolation as follows. The right lung was homogenized in Precellys tubes, then frozen in a −80 °C freezer. After thawing, the tubes were centrifuged at 12,000 x *g* at 4 °C for 5 min, and supernatants were collected for virus titration. The pellets were lysed in 1 ml Trizol for RNA isolation according to the manufacturer’s instructions.
3. Infection of mice for single cell RNA sequencing of lung immune cells. Five- to seven-week-old K18 hACE2 transgenic mice (4 mice per group, total of 16 mice) were either mock-infected with medium or infected as described above. On day 3 mice were euthanized as described above, and the lungs were isolated for immune cell isolation.

### Single cell isolation and RNA sequencing

Freshly collected whole lungs were dissected into single lobes to isolate single cells using a mouse lung dissociation kit (Miltenyi Biotec) and a gentleMACS Dissociator (Miltenyi Biotec), and red blood cells were lysed with 1X RBC lysis buffer (eBioscience). Cell suspensions were processed for single-cell sequencing following the protocol for Chromium Next GEM Single Cell 5′ Version 2 (dual index). 10,000 cells were targeted. The transcriptome of each cell was indexed with a pool of 750,000 barcodes by partitioning each cell into Gel beads-in-emulsion (GEMs) combined with a Master Mix containing reverse transcription (RT) reagents and poly(dT) RT primers. The emulsion was generated using Next GEM chips and the Chromium Controller device (10x Genomics). The RT reaction to the generated emulsion produced 10x barcoded full-length cDNA from poly-adenylated mRNA. This initial cDNA was PCR amplified to produce material for 5′ gene expression sequencing. After PCR amplification, bioanalyzer quality control was performed for all the samples using the Agilent Bioanalyzer High Sensitivity DNA assay in the 2,100 expert software (Agilent). All the samples passed the initial quality control with a cDNA size 700−1,500 bp.

Amplified full-length cDNA from polyadenylated mRNA was used to generate 5′ gene expression (GEX) libraries. The cDNA was enzymatically fragmented, and size-selected to optimize the cDNA amplicon size. P5, P7, i5, and i7 sample indexes, and Illumina R2 sequences (read 2 primer sequences) were added via End Repair, A-tailing, Adaptor ligation, and sample index PCR. A second quality control was performed for each library before sequencing, with an expected library size 500–900 bp. Finally, libraries were pooled and sequenced by the New York Genome Center (NYGC) using a NovaSeq sequencer and S2 flowcell with a minimum of 20,000 read pairs per cell.

### scRNA sequencing data processing

To enable quantification of both viral and host genes from single-cell data, we first merged the mm10(GRCm38.p6) and SARS-CoV-2 reference genomes (ASM993790v1). Cell Ranger v7.0.0 (10× Genomics) was then used to demultiplex cellular barcodes and align the reads to the combined genome. The filtered feature-barcode matrix per sample was then read into a Seurat object [113]. Putative doublets from each sample were identified using scds [114]. Any cell with the scds hybrid doublet score of 0.8 (doublet scoring based on co-expression and binary classification) was considered a putative doublet and was removed. Additionally, cells with unusually high expression levels (nFeature over 4,000 or nCount over 10,000) were also considered putative doublets and were removed from further analysis. Poor quality cells (nFeature of less than 1,000 or over 5% mitochondrial content) were also filtered out. The merged Seurat dataset was examined for the presence of intrinsic batches. All the samples were then integrated following the Seurat integration pipeline while correcting for the identified batch labels.

Initial cell-type annotation was performed with SingleR using the reference from the Immunologic Genome Project, accessed via celldex [115]. Cell cycle (G2M and S phases) scores were calculated using the Seurat function CellCycleScoring. A single cluster with high G2M and S phase scores was identified and labeled as “Proliferating”. Putative cell type annotation of each cluster was also validated using canonical marker expression (Figs 8C and S7). Major clusters corresponding to 15 cell types were identified among single cells which were isolated from the lung tissue. The average expression of SARS-CoV-2 viral genes in each cell type/sample pair was also calculated (Fig 9).

Within each cell type, differential expression analysis was performed between the following sample groups: WT versus mock, NSP1 mutant versus WT, and NSP15 mutant versus WT. Differentially expressed genes (DEG) at the single-cell level were identified using the Wilcoxon test. Those passing the FDR-adjusted *p*-value < 0.05 and absolute log2 fold change > 0.3 in expression were selected as significant. We also performed a DEG analysis at the pseudo-bulk per cell-type level. Because of the small sample sizes, this analysis did not have sufficient power to detect differentially expressed genes and was not used further.

Pathway enrichment analysis was performed on the sets of DEG with EnrichR [116] using the Gene Ontology biological process terms. All terms with FDR-adjusted *p*-value < 0.1 were considered significant. Since Gene Ontology is a hierarchy with closely related terms that correspond to highly overlapping gene sets, we sub-selected significant terms for presentation in Figs 10A, 10B, and S10. The sub-selection was done as follows: terms were included if there was a difference of at least two regulated genes from every other term. The direction of regulation for each enriched term was determined by the proportion of upregulated DEG versus the downregulated DEG for each cell type.

### Integration of the nongenomic data in the heat map

The heatmap (S13 Fig) depicts the overall changes in multiple parameters for all SARS-CoV-2 mutants. The mean of individual values of plaque size, IFN-I signaling with and without IFN-α treatment, expression of DC maturation markers CD80, CD86, and MHC-II were calculated for each mutant, followed by normalization to the mean values for WT SARS-CoV-2. In addition, for the two mutants, NSP1 and NSP15, quantitative data for several additional parameters, including mice survival, mice weight, viral load in the nasal turbinates and the lungs and pathology scores were included. The heatmap was generated through GraphPad Prism (version 9.5.1).

### Statistical analysis

Statistical analyses and generation of graphs were performed using GraphPad Prism version 10. One-way Anova or two-way ANOVA followed by Tukey’s multiple comparisons test, log-rank (Mantel–Cox) test, and pairwise *t* test (as indicated in figure legends) were used for the determination of statistical significance.

## Supporting information

S1 Fig Verification of the mutation(s) in SARS-COV-2 mutant strains by Sanger DNA sequencing.
The histograms and their sequences for the mutated region in the mutants (the bottom rows) are shown and compared with their corresponding regions in the WT virus (the top rows).
(PDF)

S2 Fig Growth kinetics of the 12 mutated viruses.
Differences for each mutant compared to WT analyzed by one way ANOVA, *p < 0.05, **p < 0.01, ***<0.001, ****p < 0.0001. **A.** Calu-3 cells, MOI 0.01 PFU/cell, panel of 12 viruses and WT SARS-CoV-2. Mean values based on duplicate samples ± SEM. **B.** WT and the NSP1 and NSP15 mutants selected for in-depth investigation in Vero-AT cells and A549-hACE-2 cells, MOI 0.01 PFU/cell. Mean values of triplicate samples ± SEM. The data underlying panels A and B in this Figure can be found in S1 Data.
(PDF)

S3 Fig Quantification of cells positive for the viral antigen using immunofluorescent microscopy.
293T-ACE2/TMPRSS2 cells were mock-infected or infected with WT SARS-CoV-2 or its mutants at an MOI of 0.3 PFU/cell. The plaques were immunostained with rabbit immune serum specific for SARS CoV-2 NSP3 (red), and the nuclei were stained with DAPI (blue). Mean percentages of virus-positive cells ± SD based on biological triplicates are shown.
(PDF)

S4 Fig SARS-CoV-2 NSP1 and NSP15 suppress pathways involved in innate immune response.
**A**, **B**. Pathways upregulated (**A**) and down-regulated (**B**) in SARS-CoV-2 NSP1 mutant-infected Calu-3 cells in comparison to WT virus infection. The P value color legend is the same for panels A and B. **C**, **D**. Pathways upregulated (C) and down-regulated (D) in SARS-CoV-2 NSP15 mutant-infected Calu-3 cells in comparison to WT virus infection. The data underlying this Figure can be found in GEO database, accession number GSE254699.
(PDF)

S5 Fig Gene expression in Calu-3 cells infected with WT or NSP-1 or NSP-15 SARS-CoV-2 mutants or mock-infected assessed by RNA-seq.
Gene expression of four ISGs with the lowest variance, IFNB1, CCL4, MX1, and IFIT1 normalized to viral reads in triplicates (**A**), and expression of selected viral genes (S, ORF1ab, ORF3a, ORF7a, ORF7b, ORF8, ORF10, and M) in triplicates across different sample groups (**B**). Boxplots represent expression values of ISGs selected based on low variance normalized to viral reads (A) and viral reads specific to 8 different viral genes (B) in four experimental conditions: mock, WT, NSP1, and NSP15. Expression values were measured using transcriptomic analysis and are displayed for each gene category (ISG and viral reads) across the four sample groups. Each boxplot summarizes the distribution of host mRNA or viral read levels, with jittered points representing individual ISGs across all three replicates, for better visualization of the underlying data distribution. Panel A shows that the mutations in NSP1 or NSP15 enhance expression of ISGs, and panel B shows that the mutations reduce the number of viral RNA reads. See Methods for details. The data underlying this Figure can be found in GEO database, accession number GSE254699.
(PDF)

S6 Fig Bulk RNA-seq for transcriptome analysis of lungs in hACE2 transgenic mice infected by SARS-CoV-2 NSP1 and NSP15 mutants on day 4: comparison with WT-infected mice.
**A.** Heat map analysis of DEG between WT and NSP1 mutant viruses. **B.** Heat map analysis of DEG between WT and NSP15 mutant viruses. The data underlying this Figure can be found in GEO database, accession number GSE254969.
(PDF)

S7 Fig Violin plots showing gene expression of selected markers for interstitial macrophages (1st row) and monocytes (2nd row) in the IM/Monocyte population, separated by groups (mock, WT, NSP1 and NSP15).
The data underlying this Figure can be found in GEO database, accession number GSE 255483. The data underlying this Figure can be found in GEO database, accession number GSE 255483.
(PDF)

S8 Fig Viral reads in individual populations of immune cells isolated from mice infected with WT or mutated SARS-CoV-2.
Dot plots showing the average expression of SARS-CoV-2 viral gene N (**A**) and ORF1AB (**B**) in each cell type/sample pair. Cell types with fewer than 20 cells in each sample are excluded from the plot. The value is not displayed in the plot (i.e., set to zero) for any cell type/sample pair that has fewer than 20 cells.
(PDF)

S9 Fig Compact volcano plots showing scRNAseq differential expression across major cell populations.
Upregulated genes are defined as those with FDR adjusted *p*-value < 0.05 and log2FC > 0.3 and are represented by orange dots. Downregulated genes are defined as those with FDR adjusted *p*-value < 0.05 and log2FC < −0.3 and are represented by purple dots. Genes with adjusted *p*-value < 0.05 and log2FC between −0.3 and 0.3 are represented by gray dots. All *p*-values are calculated using the Wilcoxon Rank Sum test and adjusted for multiple testing correction (BH). Numbers of upregulated and downregulated genes per cell type are displayed in parentheses. **A.** Comparison for WT SARS-CoV-2 versus mock. **B.** Comparison for NSP1 mutant versus WT. **C.** Comparison for NSP15 mutant versus WT. The data underlying this Figure can be found in GEO database, accession number GSE 255483.
(PDF)

S10 Fig Dot plot showing GO terms from pathway enrichment analyses by EnrichR among differentially expressed genes (DEG) for the WT versus mock.
Dot size represents the fraction of DEG within the GO term. Dot color represents the direction of the regulation of the term in the corresponding cell type (up-regulation: yellow; down-regulation: purple) and the color scale indicates the adjusted p-value (shown are only terms with FDR-adjusted enrichment *p*-value < 0.1). Terms with substantial gene overlap are filtered out, with terms remaining only if there is a difference of at least two regulated genes from every other term. The direction of regulation for each enriched term is determined by the proportion of upregulated DEG versus the downregulated DEG across all cell types. The data underlying this Figure can be found in GEO database, accession number GSE 255483.
(PDF)

S11 Fig Activation of DCs following infection with WT SARS-CoV-2, NSP1 mutant, or NSP15 mutant assessed by single-cell sequencing.
**A.** Violin plots showing expression levels of markers of DC activation CD80 and CD86. **B.** Violin plots showing expression levels of IFN-I inducible genes ISG15 and OAS1g. The data underlying this Figure can be found in GEO database, accession number GSE 255483.
(PDF)

S12 Fig Activation of DC subpopulations characterized by single-cell sequencing.
**A.** Integrated re-clustered UMAP of all mock (*n* = 3), WT (*n* = 4), NSP1 mutant (*n* = 4), and NSP15 mutant (*n* = 4) cells in the DC population colored by cluster number (top) and DC subtype (bottom, based on markers in panel C). **B.** Dot plot showing canonical marker expression in the DC sub-clusters. Relevant marker sets for each DC sub-population are highlighted in the same color as the corresponding sub-populations in the UMAP (B, bottom). **C.** Box plots comparing cell-type proportions of the DC sub-populations observed in Mock, WT, NSP1, and NSP15 groups. The limits of the box reflect the interquartile range (IQR: Q3–Q1) with the medians shown as horizontal bars. Whiskers extend to 1.5 times the IQR of the box. For each cell type, pairwise *t* test comparisons of the wild type (WT) proportion with every other group are shown (*p-value < 0.05, ***p-value < 0.001, ns - nonsignificant). The data underlying this Figure can be found in GEO database, accession number GSE 255483.
(PDF)

S13 Fig Contribution of individual SARS-CoV-2 proteins in viral immunosuppression and pathogenicity.
Summary of the effects of the 12 mutated proteins on the viral phenotype and innate and adaptive immune responses shown in Figs 1, 2, 3, and 5. The darker colors reflect the greater effects of the mutations on the biological effects indicated at the left, as compared to WT SARS-CoV-2, and therefore a greater contribution of the corresponding proteins in these biological effects. The transcriptional effects in human cells and in mice are summarized in Figs 4, 6, 7, and 9 and are not included in this heat map. The data underlying this Figure can be found in S2 Data.
(PDF)

S1 Table Primers used for mutagenesis.
(PDF)

S2 Table Criteria used for histopathology scoring.
(PDF)

S3 Table Differentially expressed genes in mice infected with WT SARS-CoV-2.
(PDF)

S1 Data Quantitative data underlying Figs 2B, 2C, 3C, 5B, 6B, 6C, 6E, 6G, 6I, and S2A, S2B.
(XLSX)

S2 Data Quantitative data underlying S13 Fig.
(XLSX)

## Abbreviations

3CLpro: 3C-like protease
ABSL-3: animal biosafety level 3
ACE2: angiotensin-converting enzyme 2
ARDS: acute respiratory distress syndrome
DCs: dendritic cells
DEG: Differentially expressed genes
FC: fold change
GEX: gene expression
GEMs: Gel beads-in-emulsion
hACE-2: human angiotensin receptor 2
IFN: Interferons
IFN-I: I IFNs
LPS: lipopolysaccharide
MEM: minimal essential medium
MFI: mean fluorescent intensity
NOD: nucleoside-binding oligomerization domain
NYGC: New York Genome Center
ORF: open reading frames
PBMC: peripheral blood mononuclear cells
PCA: Principal component analysis
PFU: plaque-forming units
PLpro: papain-like protease
PSG: penicillin-streptomycin-glutamine
PBS: phosphate buffered saline
rRNA: ribosomal RNA
RT: reverse transcription
TMPRSS2: transmembrane serine protease 2
UMI: unique molecular identification
VOC: variants of concern
WT: wild-type.
