# Supplementary material for: Redundant and distinct mechanisms suppress innate immune activation during SARS-CoV-2 infection
Source: PLoS Biol. 2026 May 20;24(5):e3003808. doi: 10.1371/journal.pbio.3003808 (PMC13221149; doi:10.1371/journal.pbio.3003808)
Supplement: S1 Fig — Verification of the mutation(s) in SARS-COV-2 mutant strains by Sanger DNA sequencing. The histograms and their sequences for the mutated region in the mutants (the bottom rows) are shown and compared with their corresponding regions in the WT virus (the top rows). (PDF) [file pbio.3003808.s001.pdf]

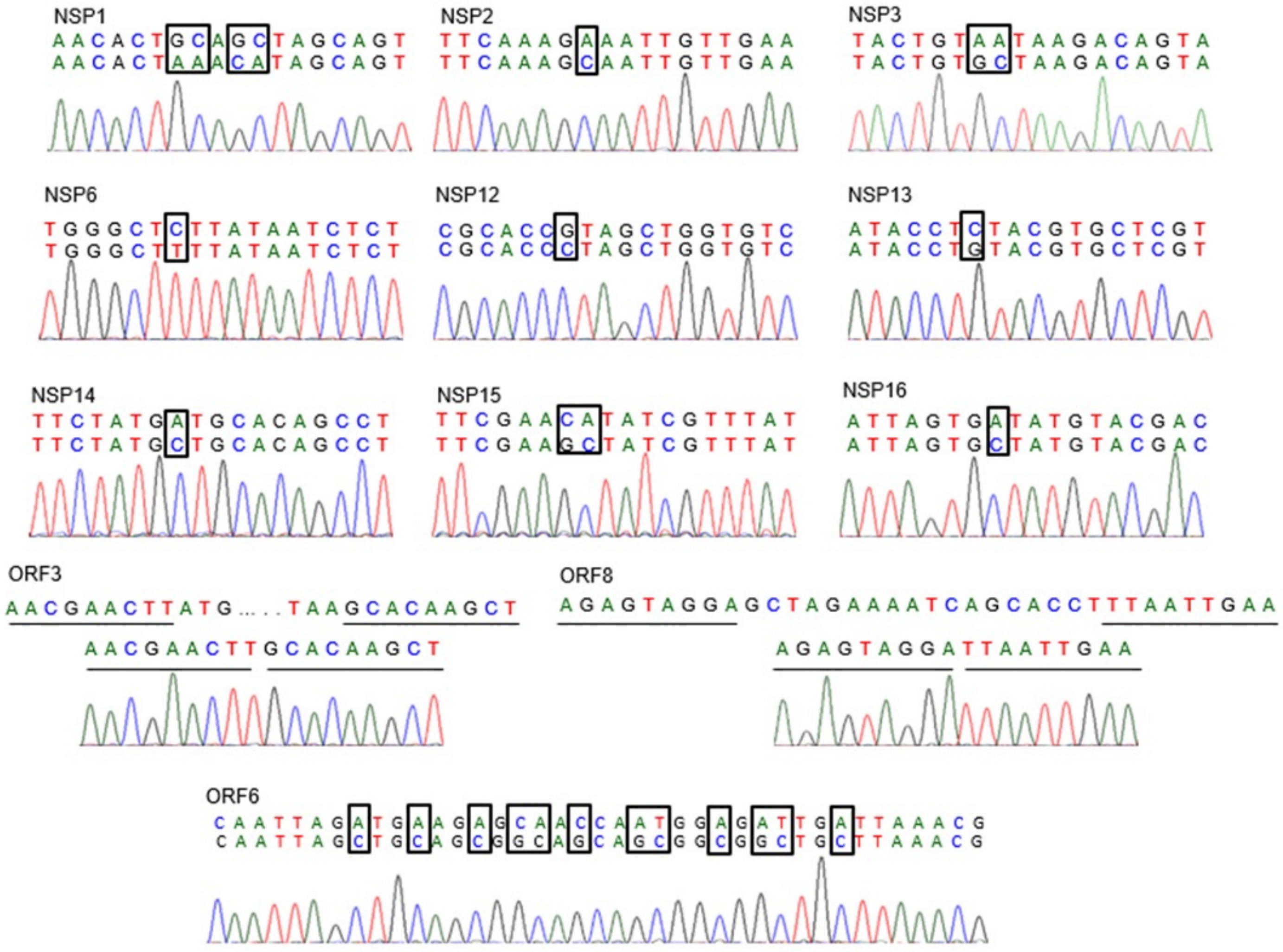

Suppl. Fig. 1. Verification of the mutation(s) in SARS-COV-2 mutant strains by Sanger DNA sequencing. The histograms and their sequences for the mutated region in the mutants (the bottom rows) are shown and compared with their corresponding regions in the WT virus (the top rows).
