# Supplementary material for: Redundant and distinct mechanisms suppress innate immune activation during SARS-CoV-2 infection
Source: PLoS Biol. 2026 May 20;24(5):e3003808. doi: 10.1371/journal.pbio.3003808 (PMC13221149; doi:10.1371/journal.pbio.3003808)
Supplement: S2 Fig — Growth kinetics of the 12 mutated viruses. Differences for each mutant compared to WT analyzed by one way ANOVA, *p < 0.05, **p < 0.01, ***<0.001, ****p < 0.0001. A. Calu-3 cells, MOI 0.01 PFU/cell, panel of 12 viruses and WT SARS-CoV-2. Mean values based on duplicate samples ± SEM. B. WT and the NSP1 and NSP15 mutants selected for in-depth investigation in Vero-AT cells and A549-hACE-2 cells, MOI 0.01 PFU/cell. Mean values of triplicate samples ± SEM. The data underlying panels A and B in this Figure can be found in S1 Data. (PDF) [file pbio.3003808.s002.pdf]

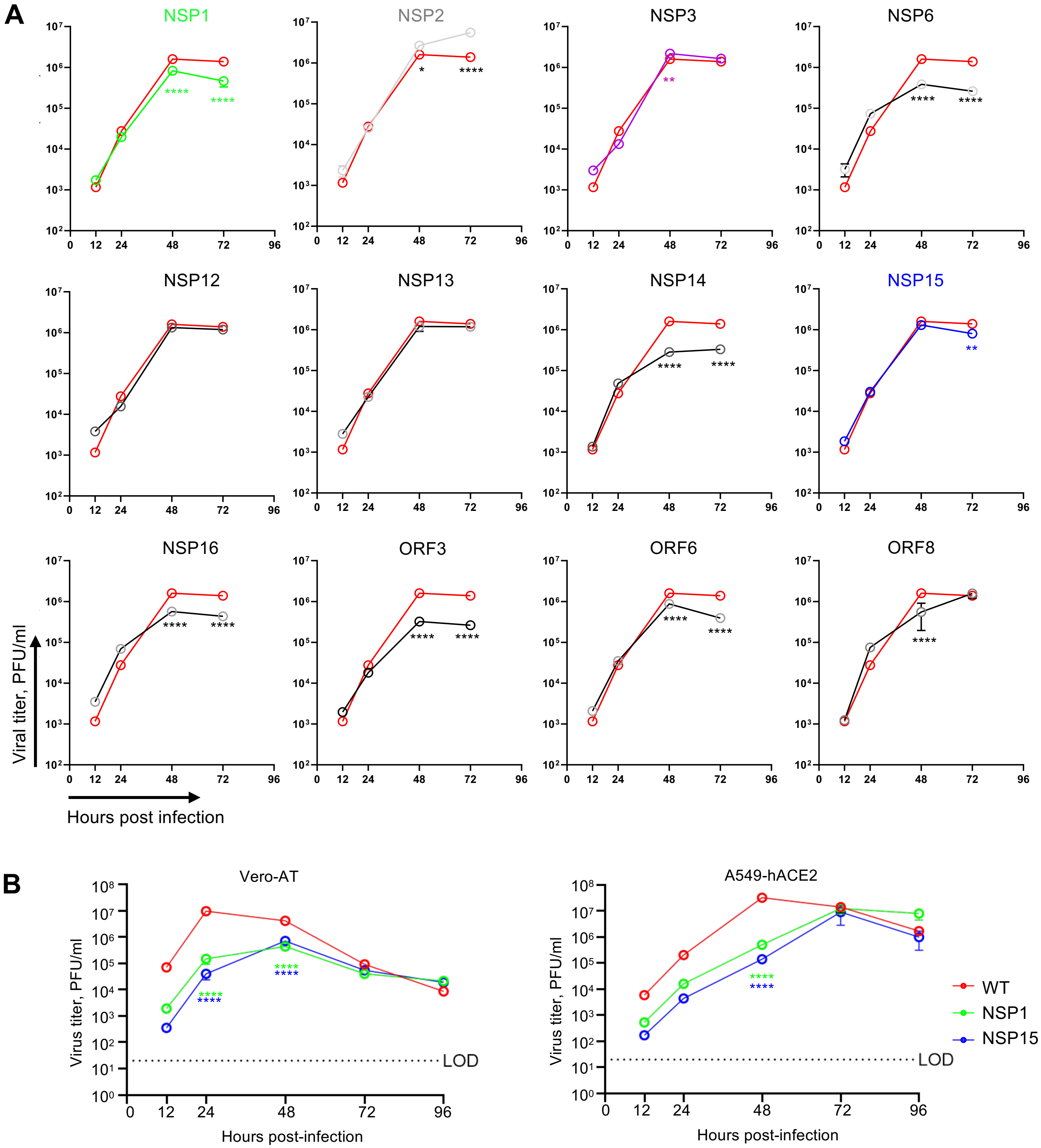

Suppl. Fig. 2. Growth kinetics of the twelve mutated viruses. Differences for each mutant compared to WT analyzed by one way ANOVA, * $p < 0.05$, ** $p < 0.01$, ***< 0.001, **** $p < 0.0001$.
A. Calu-3 cells, MOI 0.01 PFU/cell, panel of 12 viruses and WT SARS-CoV-2. Mean values based on duplicate samples ± SEM.
B. WT and the NSP1 and NSP15 mutants selected for in-depth investigation in Vero-AT cells and A549-hACE-2 cells, MOI 0.01 PFU/cell. Mean values of triplicate samples ± SEM.
The data underlying panels A and B this Figure can be found in S1 Data.
