# Supplementary material for: Redundant and distinct mechanisms suppress innate immune activation during SARS-CoV-2 infection
Source: PLoS Biol. 2026 May 20;24(5):e3003808. doi: 10.1371/journal.pbio.3003808 (PMC13221149; doi:10.1371/journal.pbio.3003808)
Supplement: S3 Fig — Quantification of cells positive for the viral antigen using immunofluorescent microscopy. 293T-ACE2/TMPRSS2 cells were mock-infected or infected with WT SARS-CoV-2 or its mutants at an MOI of 0.3 PFU/cell. The plaques were immunostained with rabbit immune serum specific for SARS CoV-2 NSP3 (red), and the nuclei were stained with DAPI (blue). Mean percentages of virus-positive cells ± SD based on biological triplicates are shown. (PDF) [file pbio.3003808.s003.pdf]

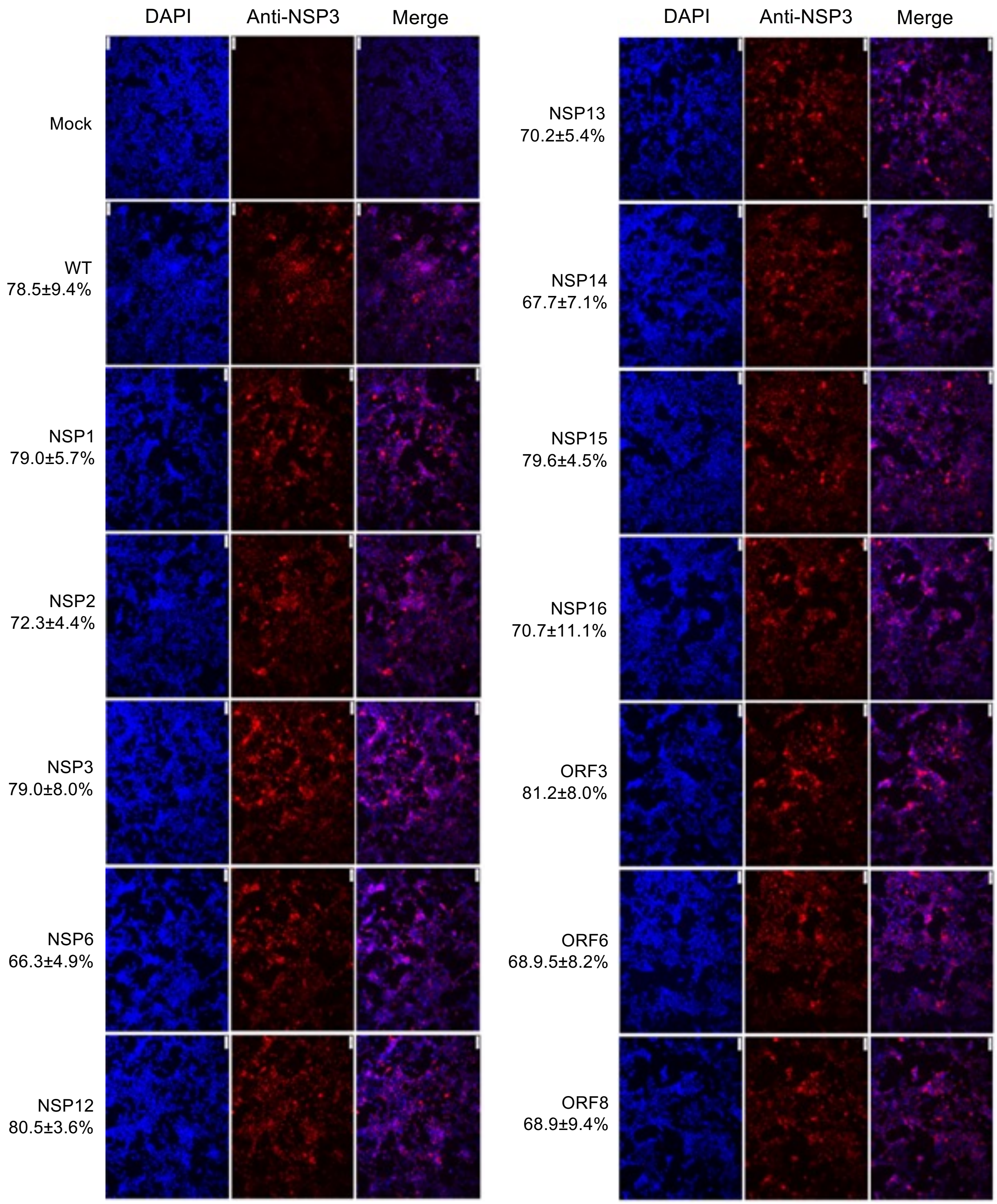

Suppl. Fig. 3. Quantification of cells positive for the viral antigen using immunofluorescent microscopy. 293T-ACE2/TMPRSS2 cells were mock-infected or infected with WT SARS-CoV-2 or its mutants at an MOI of 0.3 PFU/cell. The plaques were immunostained with rabbit immune serum specific for SARS CoV-2 NSP3 (red), and the nuclei were stained with DAPI (blue). Mean percentages of virus-positive cells ± SD based on biological triplicates are shown. Scale bar (top right in each photo): 100 μm.
