# Supplementary material for: Redundant and distinct mechanisms suppress innate immune activation during SARS-CoV-2 infection
Source: PLoS Biol. 2026 May 20;24(5):e3003808. doi: 10.1371/journal.pbio.3003808 (PMC13221149; doi:10.1371/journal.pbio.3003808)
Supplement: S4 Fig — SARS-CoV-2 NSP1 and NSP15 suppress pathways involved in innate immune response. A, B. Pathways upregulated (A) and down-regulated (B) in SARS-CoV-2 NSP1 mutant-infected Calu-3 cells in comparison to WT virus infection. The P value color legend is the same for panels A and B. C, D. Pathways upregulated (C) and down-regulated (D) in SARS-CoV-2 NSP15 mutant-infected Calu-3 cells in comparison to WT virus infection. The data underlying this Figure can be found in GEO database, accession number GSE254699. (PDF) [file pbio.3003808.s004.pdf]

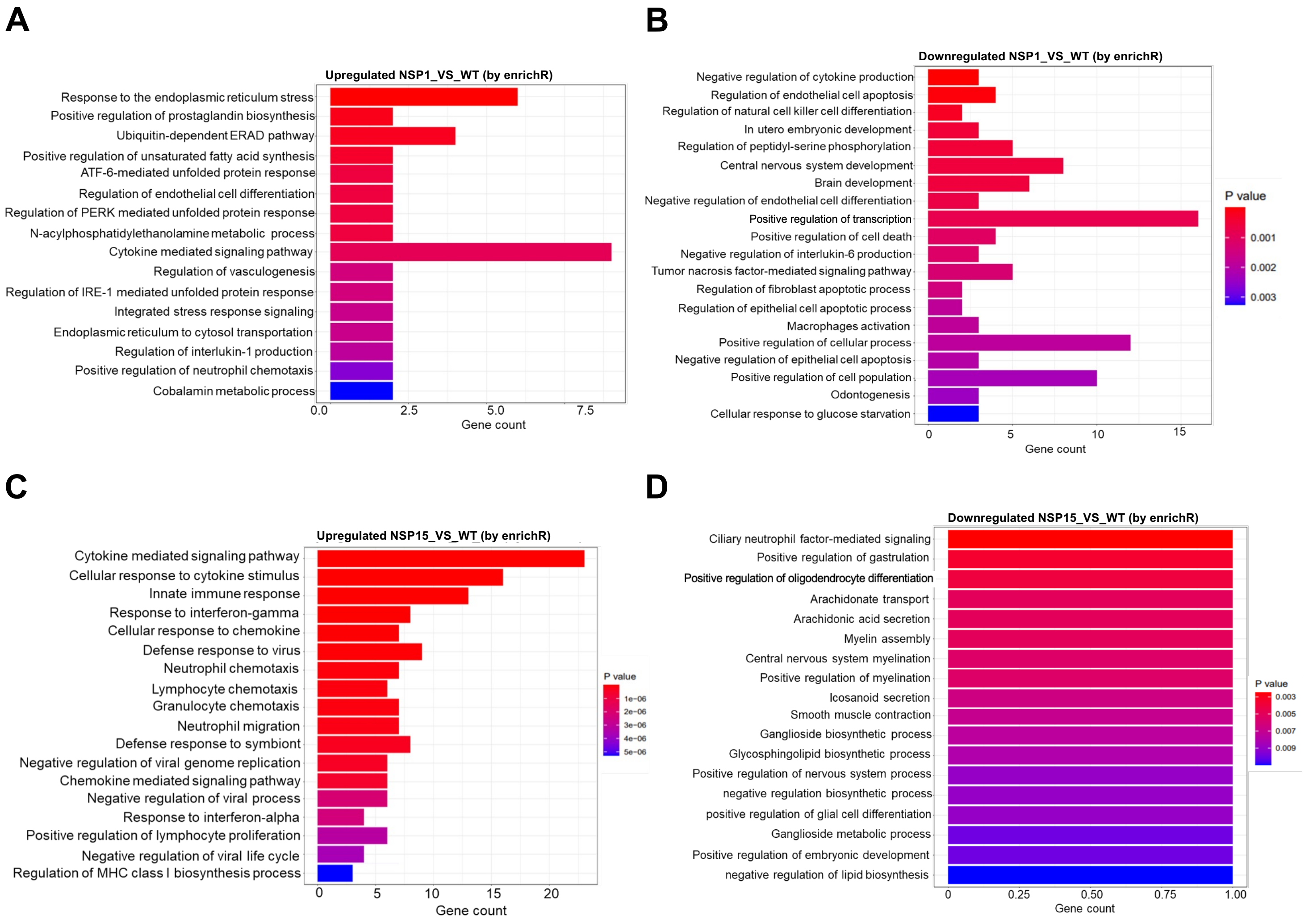

Suppl. Fig. 4. SARS-CoV-2 NSP1 and NSP15 suppress pathways involved in innate immune response.

A, **B**. Pathways upregulated (**A**) and down-regulated (**B**) in SARS-CoV-2 NSP1 mutant-infected Calu-3 cells in comparison to WT virus infection. The P value color legend is the same for panels A and B.

C, **D**. Pathways upregulated (**C**) and down-regulated (**D**) in SARS-CoV-2 NSP15 mutant-infected Calu-3 cells in comparison to WT virus infection.

The data underlying this Figure can be found in GEO database, accession number GSE254699.
