# Supplementary material for: Redundant and distinct mechanisms suppress innate immune activation during SARS-CoV-2 infection
Source: PLoS Biol. 2026 May 20;24(5):e3003808. doi: 10.1371/journal.pbio.3003808 (PMC13221149; doi:10.1371/journal.pbio.3003808)
Supplement: S5 Fig — Gene expression in Calu-3 cells infected with WT or NSP-1 or NSP-15 SARS-CoV-2 mutants or mock-infected assessed by RNA-seq. Gene expression of four ISGs with the lowest variance, IFNB1, CCL4, MX1, and IFIT1 normalized to viral reads in triplicates (A), and expression of selected viral genes (S, ORF1ab, ORF3a, ORF7a, ORF7b, ORF8, ORF10, and M) in triplicates across different sample groups (B). Boxplots represent expression values of ISGs selected based on low variance normalized to viral reads (A) and viral reads specific to 8 different viral genes (B) in four experimental conditions: mock, WT, NSP1, and NSP15. Expression values were measured using transcriptomic analysis and are displayed for each gene category (ISG and viral reads) across the four sample groups. Each boxplot summarizes the distribution of host mRNA or viral read levels, with jittered points representing individual ISGs across all three replicates, for better visualization of the underlying data distribution. Panel A shows that the mutations in NSP1 or NSP15 enhance expression of ISGs, and panel B shows that the mutations reduce the number of viral RNA reads. See Methods for details. The data underlying this Figure can be found in GEO database, accession number GSE254699. (PDF) [file pbio.3003808.s005.pdf]

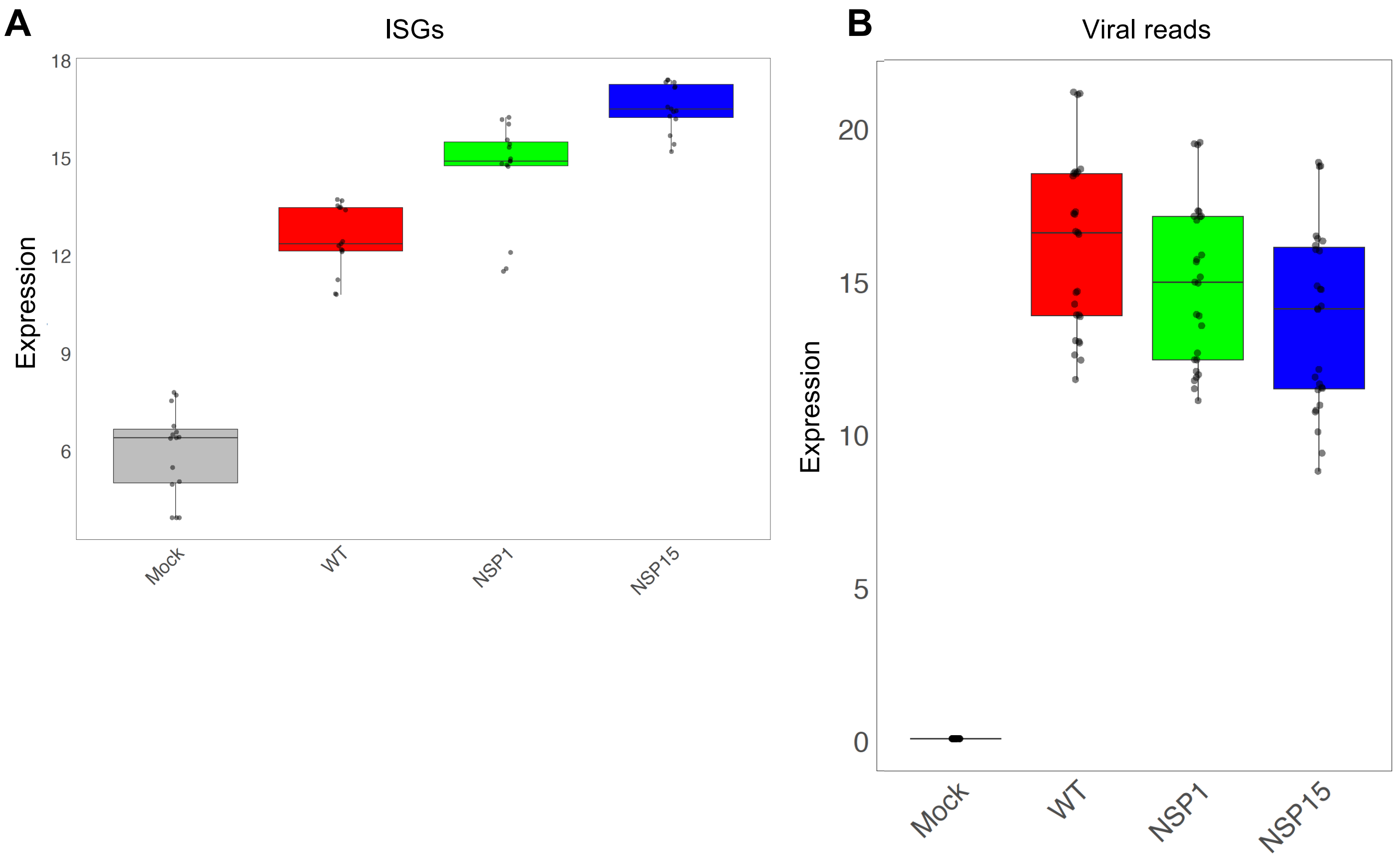

Suppl. Fig. 5. Gene expression in Calu-3 cells infected with WT or NSP-1 or NSP-15 SARS-CoV-2 mutants or mock-infected assessed by RNA-seq. Gene expression of four ISGs with the lowest variance, IFNB1, CCL4, MX1, IFIT1 normalized to viral reads in triplicates (**A**) and expression of selected viral genes (S, ORF1ab, ORF3a, ORF7a, ORF7b, ORF8, ORF10, M) in triplicates across different sample groups (**B**). Boxplots represent expression values of ISGs selected based on low variance normalized to viral reads (A) and viral reads specific to 8 different viral genes (B) in four experimental conditions: mock, WT, NSP1, and NSP15. Expression values were measured using transcriptomic analysis and are displayed for each gene category (ISG and viral reads) across the four sample groups. Each boxplot summarizes the distribution of host mRNA or viral read levels, with jittered points representing individual ISGs across all three replicates, for better visualization of the underlying data distribution. Panel A shows that the mutations in NSP1 or NSP15 enhance expression of ISGs, and panel B shows that the mutations reduce the number of viral RNA reads. See Methods for details. The data underlying this Figure can be found in GEO database, accession number GSE254699.
