# Supplementary figures and images for: Redundant and distinct mechanisms suppress innate immune activation during SARS-CoV-2 infection
Source: PLoS Biol. 2026 May 20;24(5):e3003808. doi: 10.1371/journal.pbio.3003808 (PMC13221149; doi:10.1371/journal.pbio.3003808)

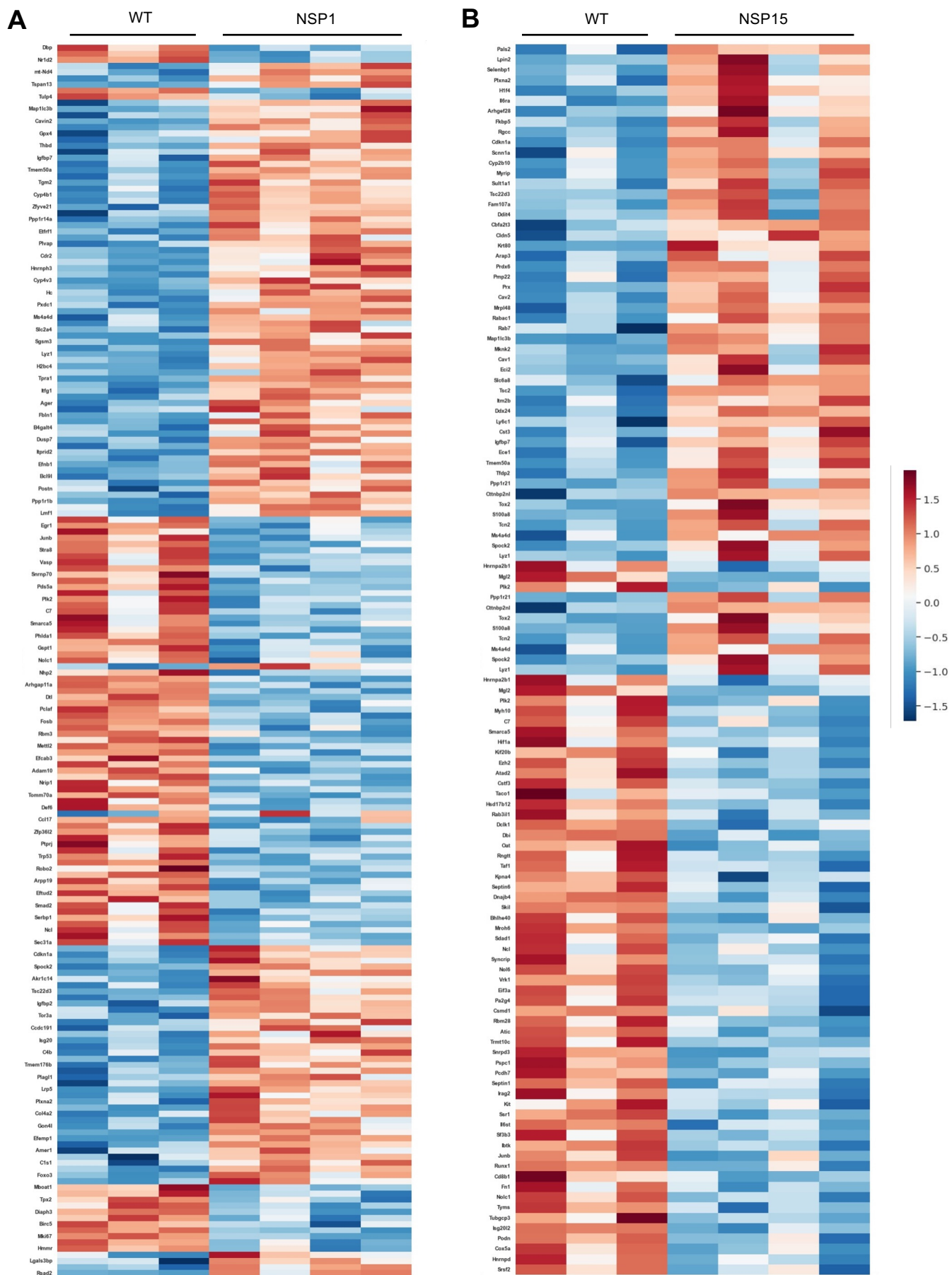

Supplement: S6 Fig — Bulk RNA-seq for transcriptome analysis of lungs in hACE2 transgenic mice infected by SARS-CoV-2 NSP1 and NSP15 mutants on day 4: comparison with WT-infected mice. A. Heat map analysis of DEG between WT and NSP1 mutant viruses. B. Heat map analysis of DEG between WT and NSP15 mutant viruses. The data underlying this Figure can be found in GEO database, accession number GSE254969. (PDF) [file pbio.3003808.s006.pdf]
