# Supplementary material for: Redundant and distinct mechanisms suppress innate immune activation during SARS-CoV-2 infection
Source: PLoS Biol. 2026 May 20;24(5):e3003808. doi: 10.1371/journal.pbio.3003808 (PMC13221149; doi:10.1371/journal.pbio.3003808)
Supplement: S7 Fig — Violin plots showing gene expression of selected markers for interstitial macrophages (1st row) and monocytes (2nd row) in the IM/Monocyte population, separated by groups (mock, WT, NSP1 and NSP15). The data underlying this Figure can be found in GEO database, accession number GSE 255483. The data underlying this Figure can be found in GEO database, accession number GSE 255483. (PDF) [file pbio.3003808.s007.pdf]

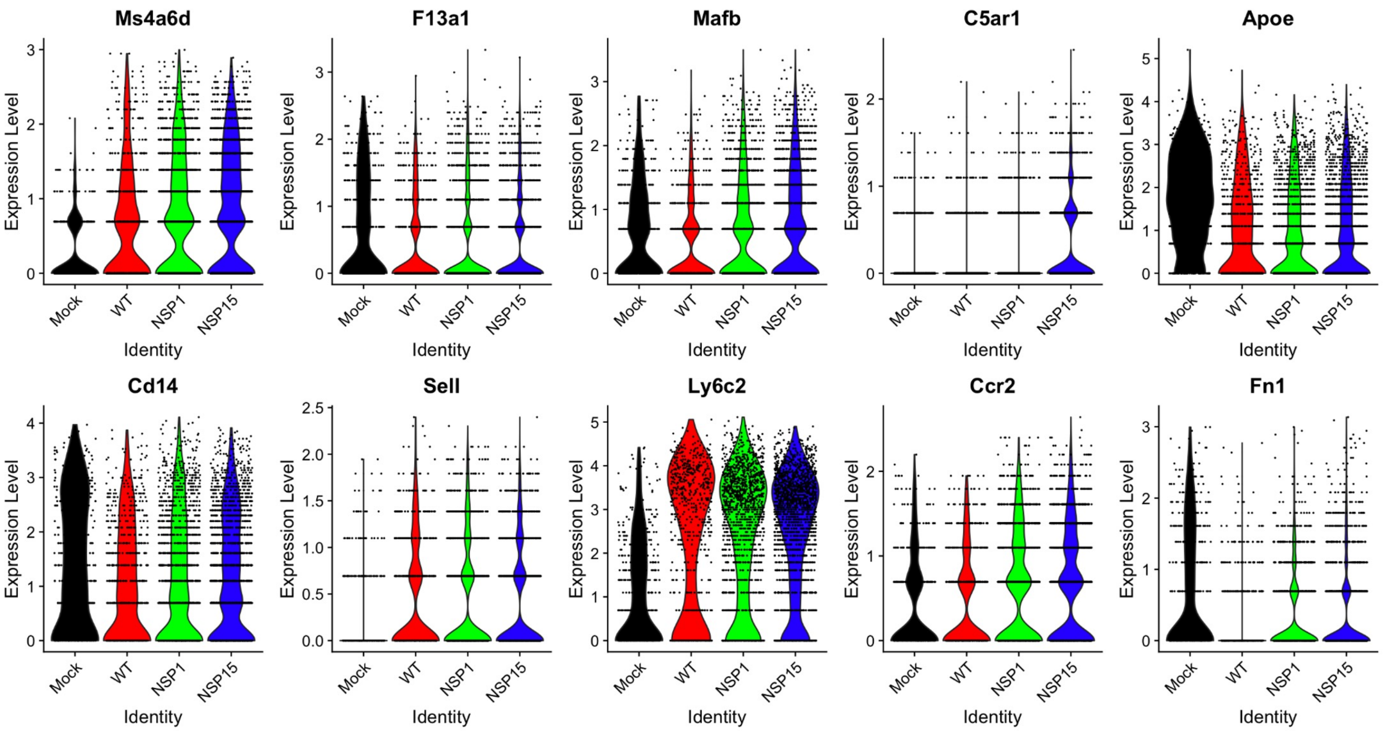

Suppl. Fig. 7. Violin plots showing gene expression of selected markers for interstitial macrophages (1st row) and monocytes (2nd row) in the IM/Monocyte population, separated by groups (mock, WT, NSP1 and NSP15). The data underlying this Figure can be found in GEO database, accession number GSE 255483.
