# Supplementary material for: Redundant and distinct mechanisms suppress innate immune activation during SARS-CoV-2 infection
Source: PLoS Biol. 2026 May 20;24(5):e3003808. doi: 10.1371/journal.pbio.3003808 (PMC13221149; doi:10.1371/journal.pbio.3003808)
Supplement: S8 Fig — Viral reads in individual populations of immune cells isolated from mice infected with WT or mutated SARS-CoV-2. Dot plots showing the average expression of SARS-CoV-2 viral gene N (A) and ORF1AB (B) in each cell type/sample pair. Cell types with fewer than 20 cells in each sample are excluded from the plot. The value is not displayed in the plot (i.e., set to zero) for any cell type/sample pair that has fewer than 20 cells. (PDF) [file pbio.3003808.s008.pdf]

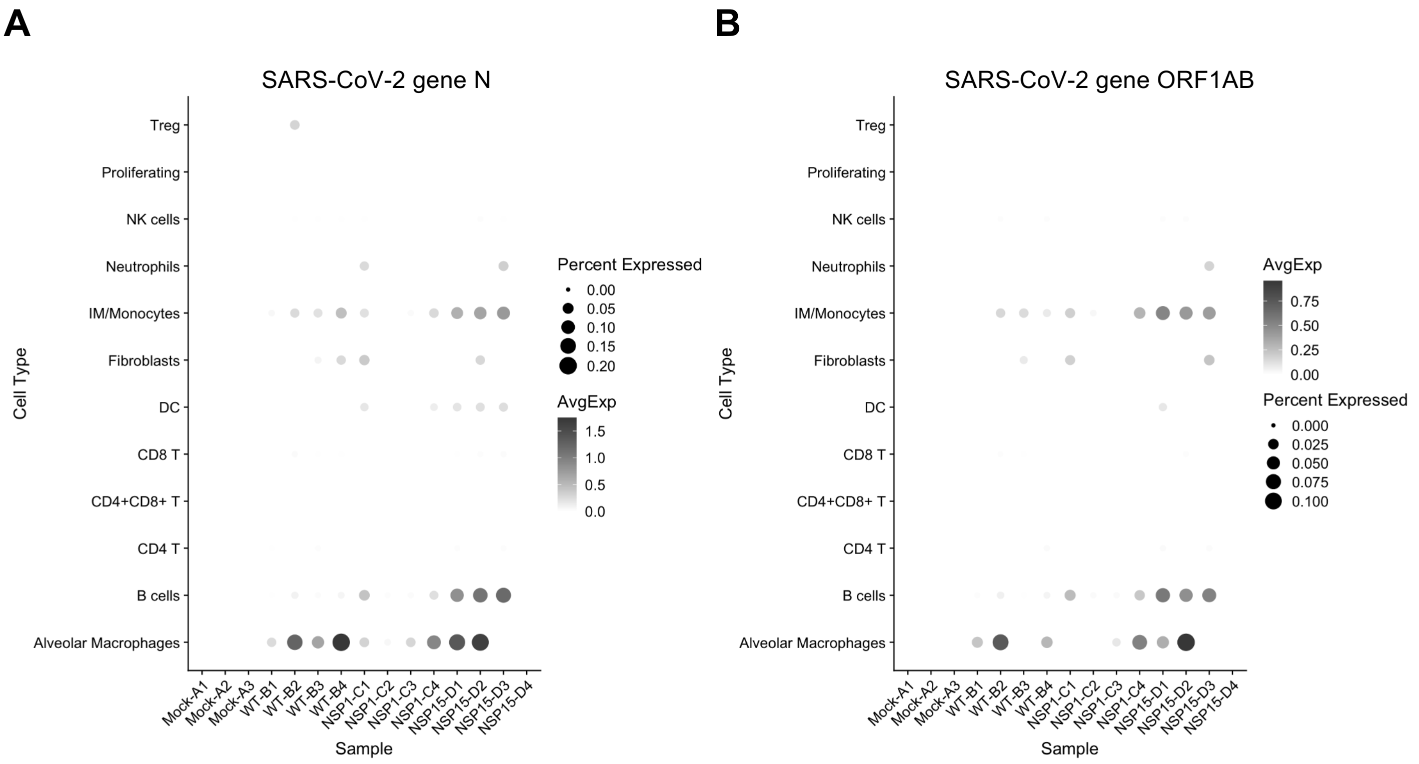

Suppl. Fig. 8. Viral reads in individual populations of immune cells isolated from mice infected with WT or mutated SARS-CoV-2. Dot plots showing the average expression of SARS-CoV-2 viral gene N (**A**) and ORF1AB (**B**) in each cell type/sample pair. Cell types with fewer than 20 cells in each sample are excluded from the plot. The value is not displayed in the plot (i.e. set to zero) for any cell type/sample pair that has fewer than 20 cells. The data underlying this Figure can be found in GEO database, accession number GSE 255483.
