# Supplementary material for: Redundant and distinct mechanisms suppress innate immune activation during SARS-CoV-2 infection
Source: PLoS Biol. 2026 May 20;24(5):e3003808. doi: 10.1371/journal.pbio.3003808 (PMC13221149; doi:10.1371/journal.pbio.3003808)
Supplement: S9 Fig — Compact volcano plots showing scRNAseq differential expression across major cell populations. Upregulated genes are defined as those with FDR adjusted p-value < 0.05 and log2FC > 0.3 and are represented by orange dots. Downregulated genes are defined as those with FDR adjusted p-value < 0.05 and log2FC < −0.3 and are represented by purple dots. Genes with adjusted p-value < 0.05 and log2FC between −0.3 and 0.3 are represented by gray dots. All p-values are calculated using the Wilcoxon Rank Sum test and adjusted for multiple testing correction (BH). Numbers of upregulated and downregulated genes per cell type are displayed in parentheses. A. Comparison for WT SARS-CoV-2 versus mock. B. Comparison for NSP1 mutant versus WT. C. Comparison for NSP15 mutant versus WT. The data underlying this Figure can be found in GEO database, accession number GSE 255483. (PDF) [file pbio.3003808.s009.pdf]

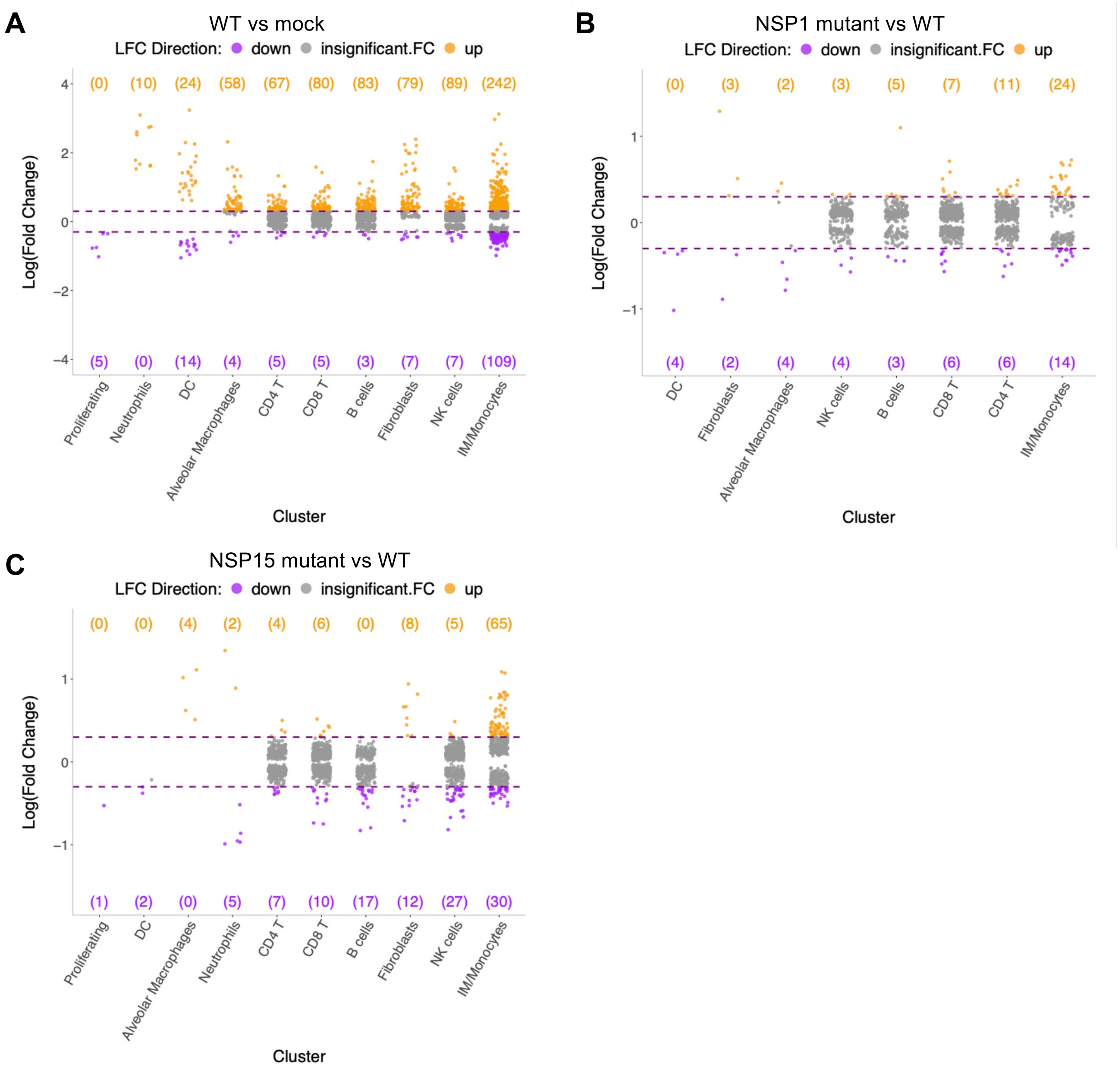

Suppl. Fig. 9. Compact volcano plots showing scRNAseq differential expression across major cell populations. Upregulated genes are defined as those with FDR adjusted p-value <0.05 and log2FC >0.3 and are represented by orange dots. Downregulated genes are defined as those with FDR adjusted p-value <0.05 and log2FC < -0.3 and are represented by purple dots. Genes with adjusted p-value <0.05 and log2FC between -0.3 and 0.3 are represented by gray dots. All p-values are calculated using the Wilcoxon Rank Sum test and adjusted for multiple testing correction (BH). Numbers of upregulated and downregulated genes per cell type are displayed in parentheses.

A. Comparison for WT SARS-CoV-2 versus mock.

B. Comparison for NSP1 mutant versus WT.

C. Comparison for NSP15 mutant versus WT.

The data underlying this Figure can be found in GEO database, accession number GSE 255483.
