# Supplementary material for: Redundant and distinct mechanisms suppress innate immune activation during SARS-CoV-2 infection
Source: PLoS Biol. 2026 May 20;24(5):e3003808. doi: 10.1371/journal.pbio.3003808 (PMC13221149; doi:10.1371/journal.pbio.3003808)
Supplement: S10 Fig — Dot plot showing GO terms from pathway enrichment analyses by EnrichR among differentially expressed genes (DEG) for the WT versus mock. Dot size represents the fraction of DEG within the GO term. Dot color represents the direction of the regulation of the term in the corresponding cell type (up-regulation: yellow; down-regulation: purple) and the color scale indicates the adjusted p-value (shown are only terms with FDR-adjusted enrichment p-value < 0.1). Terms with substantial gene overlap are filtered out, with terms remaining only if there is a difference of at least two regulated genes from every other term. The direction of regulation for each enriched term is determined by the proportion of upregulated DEG versus the downregulated DEG across all cell types. The data underlying this Figure can be found in GEO database, accession number GSE 255483. (PDF) [file pbio.3003808.s010.pdf]

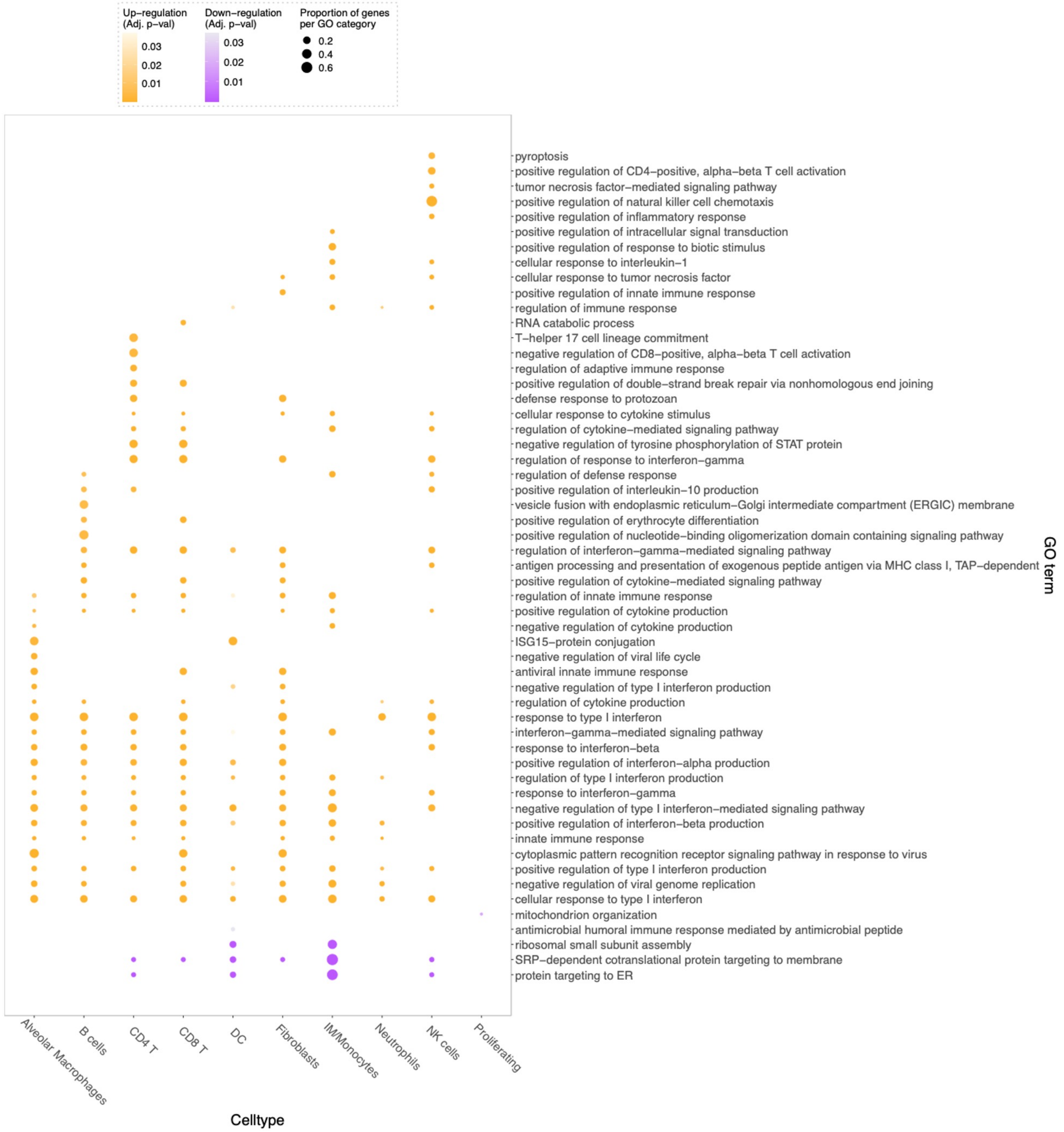


**Suppl. Fig. 10. Dot plot showing GO terms from pathway enrichment analyses by EnrichR among differentially expressed genes (DEG) for the WT vs mock.** Dot size represents the fraction of DEG within the GO term. Dot color represents the direction of the regulation of the term in the corresponding cell type (up-regulation: yellow; down-regulation: purple) and the color scale indicates the adjusted p-value (shown are only terms with FDR adjusted enrichment p-value < 0.1). Terms with substantial gene overlap are filtered out, with terms remaining only if there is a difference of at least two regulated genes from every other term. The direction of regulation for each enriched term is determined by the proportion of upregulated DEG versus the downregulated DEG across all cell types. The data underlying this Figure can be found in GEO database, accession number GSE 255483.
