# Supplementary material for: Redundant and distinct mechanisms suppress innate immune activation during SARS-CoV-2 infection
Source: PLoS Biol. 2026 May 20;24(5):e3003808. doi: 10.1371/journal.pbio.3003808 (PMC13221149; doi:10.1371/journal.pbio.3003808)
Supplement: S11 Fig — Activation of DCs following infection with WT SARS-CoV-2, NSP1 mutant, or NSP15 mutant assessed by single-cell sequencing. A. Violin plots showing expression levels of markers of DC activation CD80 and CD86. B. Violin plots showing expression levels of IFN-I inducible genes ISG15 and OAS1g. The data underlying this Figure can be found in GEO database, accession number GSE 255483. (PDF) [file pbio.3003808.s011.pdf]

A

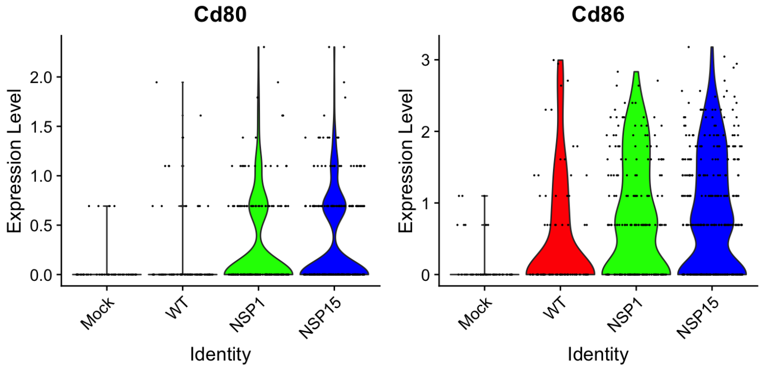

B

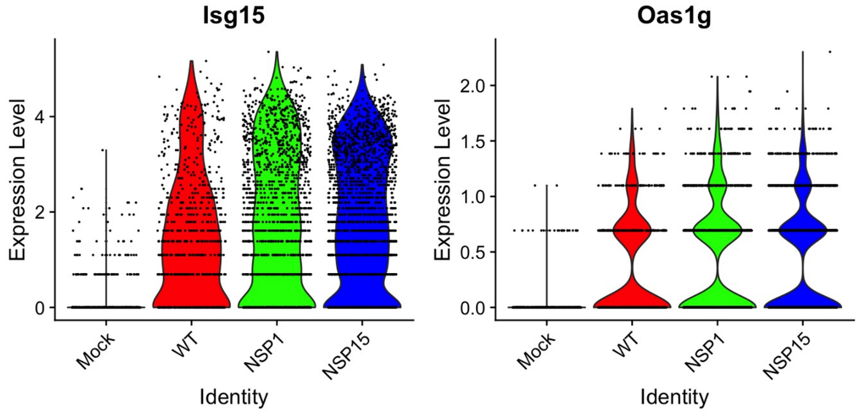

Suppl. Fig. 11. Activation of DCs following infection with WT SARS-CoV-2, NSP1 mutant or NSP15 mutant assessed by single-cell sequencing.

A. Violin plots showing expression levels of markers of DC activation CD80 and CD86.

B. Violin plots showing expression levels of IFN-I inducible genes ISG15 and OAS1g.

The data underlying this Figure can be found in GEO database, accession number GSE 255483.
