# Supplementary material for: Redundant and distinct mechanisms suppress innate immune activation during SARS-CoV-2 infection
Source: PLoS Biol. 2026 May 20;24(5):e3003808. doi: 10.1371/journal.pbio.3003808 (PMC13221149; doi:10.1371/journal.pbio.3003808)
Supplement: S13 Fig — Contribution of individual SARS-CoV-2 proteins in viral immunosuppression and pathogenicity. Summary of the effects of the 12 mutated proteins on the viral phenotype and innate and adaptive immune responses shown in Figs 1, 2, 3, and 5. The darker colors reflect the greater effects of the mutations on the biological effects indicated at the left, as compared to WT SARS-CoV-2, and therefore a greater contribution of the corresponding proteins in these biological effects. The transcriptional effects in human cells and in mice are summarized in Figs 4, 6, 7, and 9 and are not included in this heat map. The data underlying this Figure can be found in S2 Data. (PDF) [file pbio.3003808.s013.pdf]

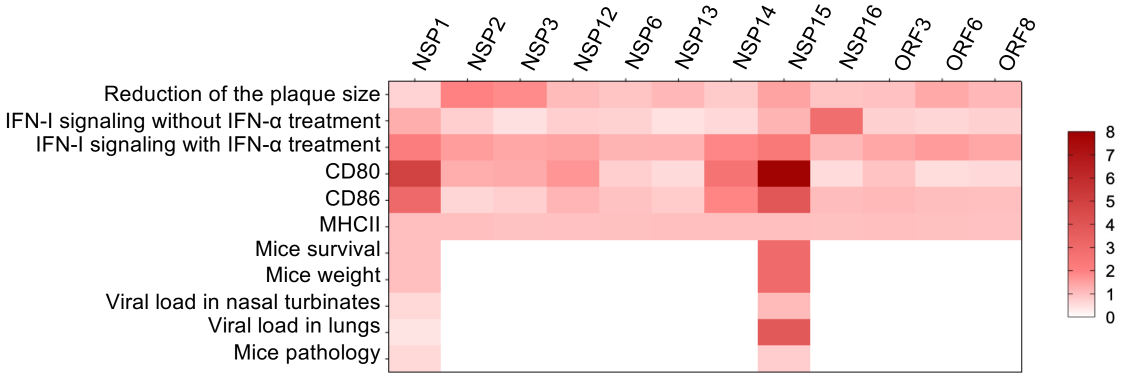

Suppl. Fig. 13. Contribution of individual SARS-CoV-2 proteins in viral immunosuppression and pathogenicity. Summary of the effects of the 12 mutated proteins on the viral phenotype and innate and adaptive immune responses shown in Fig. 1, 2, 3 and 5. The darker colors reflect the greater effects of the mutations on the biological effects indicated at the left, as compared to WT SARS-CoV-2, and therefore greater contribution of the corresponding proteins in these biological effects. The transcriptional effects in human cells and in mice are summarized in Fig. 4, 6, 7, 9 and are not included in this heat map. The data underlying this Figure can be found in S2 Data.
