# Supplementary material for: Redundant and distinct mechanisms suppress innate immune activation during SARS-CoV-2 infection
Source: PLoS Biol. 2026 May 20;24(5):e3003808. doi: 10.1371/journal.pbio.3003808 (PMC13221149; doi:10.1371/journal.pbio.3003808)
Supplement: S1 Table — Primers used for mutagenesis. (PDF) [file pbio.3003808.s014.pdf]

Supplemental Table 1. Primers used for mutagenesis

**Name**	**Sequences (5'-3')**	**Mutated gene**	**Mutation position**
NSP1M1F	acgagcttggcactgatccttatgaagattttcaagaaaactggaacactGCAGCTagcagtggtgttacccgtgaaTAGGGATAACAGGGTAATCGATTT	**NSP1**	**K164A/H165A**
NSP1M1R	tatgcccctccgttaagctcacgcatgagttcacgggtaacaccactgctAGCTGCagtgttccagttttcttgaaaatGCCAGTGTTACAACCAATTAACC		
NSP2M1F	caagtgcttttgtggaaactgtgaaaggtttggattataaagcattcaaaGCAattgttgaatcctgtggtaatttTAGGGATAACAGGGTAATCGATTT	**NSP2**	**Q321A**
NSP2M1R	tttttagcttttccttttgtaactttaaaattaccacaggattcaacaatTGCtttgaatgctttataatccaaaccGCCAGTGTTACAACCAATTAACC		
NSP3M1F	aatacccacaagttaatggtttaacttctattaaatgggcagataacaacGCTtatcttgccactgcattgttaacTAGGGATAACAGGGTAATCGATTT	**NSP3**	**C856A**
NSP3M1R	ttaaacttcaactctatttgttggagtgttaacaatgcagtggcaagataAGCgttgttatctgcccatttaatagaGCCAGTGTTACAACCAATTAACC		
NSP3M2F	caagggctggtgaagctgctaacttttgtgcacttatcttagcctactgtGCTaagacagtaggtgagttaggtgaTAGGGATAACAGGGTAATCGATTT	**NSP3**	**N901A**
NSP3M2R	aacaagtaactcattgtttctctaacatcacctaactcacctactgtcttAGCacagtaggctaagataagtgcacaGCCAGTGTTACAACCAATTAACC		
NSP3M4F	agtgtggtcactataaacatataacttctaaagaaactttgtattgcataGCCggtgctttacttacaaagtcctcTAGGGATAACAGGGTAATCGATTT	**NSP3**	**R911S/E912R**
NSP3M4R	acatccgtaataggacctttgtattctgaggactttgtaagtaaagcaccGGCtatgcaatacaaagtttctttagaGCCAGTGTTACAACCAATTAACC		
NSP3M5F	cacttatcttagcctactgtaataagacagtaggtgagttaggtgatgttAGTAGAacaatgagttacttgtttcaacTAGGGATAACAGGGTAATCGATTT	**NSP3**	**D1031A**
NSP3M5R	actcttttgcaagaatctaaattggcatgttgaaacaagtaactcattgtTCTACTaacatcacctaactcacctactGCCAGTGTTACAACCAATTAACC		
NSP5M1F	gtgctatgaggcccaatttcactattaagggttcattccttaatggttcaGCtggtagtgttggttttaacatagatTAGGGATAACAGGGTAATcgattt	**NSP5**	**C145A**
NSP5M1R	tgtaacaaaaagagacacagtcataatctatgttaaaaccaacactaccaGCtgaaccattaaggaatgaaccctGCCAGTGTTACAACCAATTAACC		
NSP6M1F	ataaagtttattatggtaatgctttagatcaagccatttccatgtgggctTtataatctctgttacttctaactTAGGGATAACAGGGTAATCGATTT	**NSP6**	**L37F**
NSP6R1R	catgacagttgtaactacacctgagtagttagaagtaacagagattataaAagcccacatggaaatggcttgatctGCCAGTGTTACAACCAATTAACC		
NSP6M2F	ttagatatatgaattcacagggactactcccacccaagaatagcatagatggcaaaccttgtatcaaagtagccaTAGGGATAACAGGGTAATCGATTT	**NSP6**	**AA268-279 deletion**
NSP6M2R	acatctgacattttagactgtacagtggctactttgatacaaggtttgccatctatgctattcttgggtgggagtGCCAGTGTTACAACCAATTAACC		
NSP12M1F	ctcaaatgaatcttaagtatgccattagtgcaaagaatagagctcgcaccCtagctggtgtctctatctgtagtaTAGGGATAACAGGGTAATCGATTT	**NSP12**	**F480L**
NSP12M1R	ttgatgaaactgtctattggtcatagtactacagatagagacaccagctaGggtgcgagctctattctttgcactaGCCAGTGTTACAACCAATTAACC		
NSP13M1F	gaaggcattaaaatatttgcctatagataaatgtagtagaattatacctgTacgtgctcgtgtagagtgttttgaTAGGGATAACAGGGTAATCGATTT	**NSP13**	**A336V**
NSP13M1R	ctaatgttgaattcactttgaatttatcaaaacactctacacgagcacgtAcaggtataattctactacatttatcGCCAGTGTTACAACCAATTAACC		
NSP14M1F	ctaaagctattaagtgtgtacctcaagctgatgtagaatggaagttctatGCTgcacagccttgtagtgacaaagcTAGGGATAACAGGGTAATCGATTT	**NSP14**	**D352A**
NSP14M1R	gaatagaataattcttctattttataagctttgtcactacaaggctgtgcAGCatagaacttccattctacatcagcGCCAGTGTTACAACCAATTAACC		
NSP15M1F	ctatggatgaattcattgaacggtataaattagaaggctatgccttcgaaGCTatcgtttatggagattttagtcaTAGGGATAACAGGGTAATCGATTT	**NSP15**	**H234A**
NSP15M1R	agtagatgtaaaccacctaactgactatgactaaaatctccataaacgatAGCttcgaaggcatagccttctaatttGCCAGTGTTACAACCAATTAACC		
NSP16M1F	gtgattgtgcaactgtacatacagctaataaatgggatctcattattagtGCTatgtacgaccctaagactaaaaaTAGGGATAACAGGGTAATcgattt	**NSP16**	**D130A**
NSP16M1R	tctttagagtcattttcttttgtaacatttttagtcttagggtcgtacatAGCactaataatgagatcccatttattGCCAGTGTTACAACCAATTAACCAATTCTGATTAG		
O3-1F	tctgagccagtgctcaaaggagtcaaattacattacacataaacgaacttgcacaagctgatgagtacgaacttaTAGGGATAACAGGGTAATCGATTT	**ORF3**	**AA1-275 deletion**
O3-1R	gtctcttccgaaacgaatgagtacataagttcgtactcatcagcttgtgcaagttcgtttatgtgtaatgtaattGCCAGTGTTACAACCAATTAACC		
O6-1F	aattaaaaatttatctaagtcactaactgagaataaatattctcaattagCtgCagCgGCaGcaGCggCgGCtgCttaaacgaacatTAGGGATAACAGGGTAATCGATTT	**ORF6**	**DEEQPMEID into AAAAAAAAA**
O6-1R	tagcgagtgttatcagtgccaagaaaagaataattttcatgttcgtttaaGcaGCcGccGCtgCtGCcGctGcaGctaattgagaataGCCAGTGTTACAACCAATTAACC		
O8-1F	ATGACCCGTGTCCTATTCACTTCTATTCTAAATGGTATATTAGAGTAGGAttaattgaattgtgcgtggatgaggTAGGGATAACAGGGTAATCGATTT	**ORF8**	**ARKSAP deletion**
O8-1R	tactgaatgggtgatttagaaccagcctcatccacgcacaattcaattaaTCCTACTCTAATATACCATTTAGAAgccagtgttacaaccaattaacc		
