# Supplementary material for: Redundant and distinct mechanisms suppress innate immune activation during SARS-CoV-2 infection
Source: PLoS Biol. 2026 May 20;24(5):e3003808. doi: 10.1371/journal.pbio.3003808 (PMC13221149; doi:10.1371/journal.pbio.3003808)
Supplement: S2 Table — Criteria used for histopathology scoring. (PDF) [file pbio.3003808.s015.pdf]

Supplemental Table 2. Criteria used for histopathology scoring

Criterium / Score	0	1	2	3	4
Extent of inflammation (% tissue involved)	0	<10	10-30	30-60	>60
Inflammatory foci type	No inflammation	Patchy inflammatory foci, few (<2)	Patchy inflammatory foci, many (>2)	Large inflammatory foci, few (<2)	Large inflammatory foci, many (>2)
Alveolar septa	Thin and delicate	Thickened in <10% HPF	Thickened in <30% HPF	Thickened in <60% HPF	Thickened in >60% HPF
Airways	Clear; no cells	Few cells in airway	Moderate cells in airway	More cells in airway; Epithelial hyperplasia	Occlusion of airway/epithelial hyperplasia or desquamation
Alveoli/ perivascular cuff/blood vessels/pleuritis/ cell types	Clear; no inflammatory cells	Few cells. Few PMN or MNC	Moderate cells/PVC/mild congestion/mild pleuritis/mostly MNC	More cells/PVC/more congestion and pleuritis/more MNC and PMN	Abundant cells/large PVC/severe congestion or pleuritis/mixed cells

The criteria were adapted from ref. 1 . HPF – high power field (>10x); PMN – polymorphonuclear cells/heterophils; MNC – mononuclear cells including lymphocytes and macrophages; PVC – perivascular cuff.

REFERENCE

1. Matute-Bello, G. et al. An official American Thoracic Society workshop report: features and measurements of experimental acute lung injury in animals. Am J Respir Cell Mol Biol 44, 725-738, doi:10.1165/rcmb.2009-0210ST (2011).
