# Supplementary material for: Redundant and distinct mechanisms suppress innate immune activation during SARS-CoV-2 infection
Source: PLoS Biol. 2026 May 20;24(5):e3003808. doi: 10.1371/journal.pbio.3003808 (PMC13221149; doi:10.1371/journal.pbio.3003808)
Supplement: S3 Table — Differentially expressed genes in mice infected with WT SARS-CoV-2. (PDF) [file pbio.3003808.s016.pdf]

Suppl. Table 3. Differentially expressed genes in mice infected with WT SARS-CoV-2

celltype	Term	Overlap	P.value	Adjusted.P.va	Old.P.value	Old.Adjusted	Odds.Ratio	Combined.Sc	Genes	Annotated	Significant	shortName	Ratio
B_cells	cellular resp	15/65	1.53E-22	5.78E-20	0	0	83.9323944	4215.96131	IFITM3;SP10	65	15	cellular resp	0.23076923
B_cells	response to i	28-May	1.19E-07	9.97E-06	0	0	53.3843264	851.257838	IFITM3;BST2;	28	5	response to i	0.17857143
B_cells	interferon-ga	Jun-68	4.68E-07	3.21E-05	0	0	24.0145161	349.993722	SP100;STAT1;	68	6	interferon-ga	0.08823529
B_cells	innate immu	10/302	6.83E-07	4.16E-05	0	0	8.84192502	125.527633	IFITM3;BST2;	302	10	innate immu	0.03311258
B_cells	response to i	Jun-80	1.23E-06	5.82E-05	0	0	20.1081081	273.579583	IFITM3;BST2;	80	6	response to i	0.075
B_cells	negative regu	May-54	3.50E-06	0.00015552	0	0	25.0251953	314.37249	IFITM3;BST2;	54	5	negative regu	0.09259259
B_cells	response to t	9-Mar	6.33E-06	0.00026545	0	0	119.927711	1435.58847	SP100;ISG15	9	3	response to t	0.33333333
B_cells	regulation of	May-64	8.16E-06	0.00030814	0	0	20.7731743	243.377286	IRF1;IFI35;PA	64	5	regulation of	0.078125
B_cells	positive regu	9/335	1.38E-05	0.00047392	0	0	7.02302605	78.5887626	ZBP1;CD274	335	9	positive regu	0.02686567
B_cells	positive regu	Apr-36	1.69E-05	0.00055467	0	0	30.3079268	333.034644	DDX58;IRF1;	36	4	positive regu	0.11111111
B_cells	regulation of	May-89	4.08E-05	0.0012467	0	0	14.5723104	147.262113	ZBP1;DDX58	89	5	regulation of	0.05617978
B_cells	negative regu	16-Mar	4.13E-05	0.0012467	0	0	55.3317887	558.579967	ISG15;SAMH	16	3	negative regu	0.1875
B_cells	positive regu	20-Mar	8.30E-05	0.0020888	0	0	42.3040397	397.517861	DDX58;STAT1	20	3	positive regu	0.15
B_cells	regulation of	23-Mar	0.00012774	0.00301393	0	0	35.9530121	322.336479	STAT1;PARP1	23	3	regulation of	0.13043478
B_cells	positive regu	5-Feb	0.00018122	0.00390922	0	0	158.02381	1361.4995	HSPA1B;HSP	5	2	positive regu	0.4
B_cells	vesicle fusio	6-Feb	0.00027107	0.00487287	0	0	118.511905	973.352544	TAP1;TAPBP	6	2	vesicle fusio	0.33333333
B_cells	positive regu	Mar-32	0.00034791	0.0059698	0	0	24.7839634	197.368809	CD274;ISG1	32	3	positive regu	0.09375
B_cells	regulation of	Apr-83	0.00045209	0.0069658	0	0	12.2476073	94.326662	ZBP1;IRF1;S	83	4	regulation of	0.04819277
DC	cellular resp	15/65	1.53E-22	5.78E-20	0	0	83.9323944	4215.96131	IFITM3;SP10	65	15	cellular resp	0.23076923
DC	response to i	28-May	1.19E-07	9.97E-06	0	0	53.3843264	851.257838	IFITM3;BST2;	28	5	response to i	0.17857143
DC	interferon-ga	Jun-68	4.68E-07	3.21E-05	0	0	24.0145161	349.993722	SP100;STAT1;	68	6	interferon-ga	0.08823529
DC	innate immu	10/302	6.83E-07	4.16E-05	0	0	8.84192502	125.527633	IFITM3;BST2;	302	10	innate immu	0.03311258
DC	response to i	Jun-80	1.23E-06	5.82E-05	0	0	20.1081081	273.579583	IFITM3;BST2;	80	6	response to i	0.075
DC	negative regu	May-54	3.50E-06	0.00015552	0	0	25.0251953	314.37249	IFITM3;BST2;	54	5	negative regu	0.09259259
DC	response to t	9-Mar	6.33E-06	0.00026545	0	0	119.927711	1435.58847	SP100;ISG15	9	3	response to t	0.33333333
DC	regulation of	May-64	8.16E-06	0.00030814	0	0	20.7731743	243.377286	IRF1;IFI35;PA	64	5	regulation of	0.078125
DC	positive regu	9/335	1.38E-05	0.00047392	0	0	7.02302605	78.5887626	ZBP1;CD274	335	9	positive regu	0.02686567
DC	positive regu	Apr-36	1.69E-05	0.00055467	0	0	30.3079268	333.034644	DDX58;IRF1;	36	4	positive regu	0.11111111
DC	regulation of	May-89	4.08E-05	0.0012467	0	0	14.5723104	147.262113	ZBP1;DDX58	89	5	regulation of	0.05617978
DC	negative regu	16-Mar	4.13E-05	0.0012467	0	0	55.3317887	558.579967	ISG15;SAMH	16	3	negative regu	0.1875
DC	positive regu	20-Mar	8.30E-05	0.0020888	0	0	42.3040397	397.517861	DDX58;STAT1	20	3	positive regu	0.15
DC	regulation of	23-Mar	0.00012774	0.00301393	0	0	35.9530121	322.336479	STAT1;PARP1	23	3	regulation of	0.13043478
DC	positive regu	5-Feb	0.00018122	0.00390922	0	0	158.02381	1361.4995	HSPA1B;HSP	5	2	positive regu	0.4
DC	vesicle fusio	6-Feb	0.00027107	0.00487287	0	0	118.511905	973.352544	TAP1;TAPBP	6	2	vesicle fusio	0.33333333
DC	positive regu	Mar-32	0.00034791	0.0059698	0	0	24.7839634	197.368809	CD274;ISG1	32	3	positive regu	0.09375
DC	regulation of	Apr-83	0.00045209	0.0069658	0	0	12.2476073	94.326662	ZBP1;IRF1;S	83	4	regulation of	0.04819277
Fibroblasts	cellular resp	15/65	1.53E-22	5.78E-20	0	0	83.9323944	4215.96131	IFITM3;SP10	65	15	cellular resp	0.23076923
Fibroblasts	response to i	28-May	1.19E-07	9.97E-06	0	0	53.3843264	851.257838	IFITM3;BST2;	28	5	response to i	0.17857143
Fibroblasts	interferon-ga	Jun-68	4.68E-07	3.21E-05	0	0	24.0145161	349.993722	SP100;STAT1;	68	6	interferon-ga	0.08823529
Fibroblasts	innate immu	10/302	6.83E-07	4.16E-05	0	0	8.84192502	125.527633	IFITM3;BST2;	302	10	innate immu	0.03311258
Fibroblasts	response to i	Jun-80	1.23E-06	5.82E-05	0	0	20.1081081	273.579583	IFITM3;BST2;	80	6	response to i	0.075
Fibroblasts	negative regu	May-54	3.50E-06	0.00015552	0	0	25.0251953	314.37249	IFITM3;BST2;	54	5	negative regu	0.09259259
Fibroblasts	response to t	9-Mar	6.33E-06	0.00026545	0	0	119.927711	1435.58847	SP100;ISG15	9	3	response to t	0.33333333
Fibroblasts	regulation of	May-64	8.16E-06	0.00030814	0	0	20.7731743	243.377286	IRF1;IFI35;PA	64	5	regulation of	0.078125
Fibroblasts	positive regu	9/335	1.38E-05	0.00047392	0	0	7.02302605	78.5887626	ZBP1;CD274	335	9	positive regu	0.02686567
Fibroblasts	positive regu	Apr-36	1.69E-05	0.00055467	0	0	30.3079268	333.034644	DDX58;IRF1;	36	4	positive regu	0.11111111
Fibroblasts	regulation of	May-89	4.08E-05	0.0012467	0	0	14.5723104	147.262113	ZBP1;DDX58	89	5	regulation of	0.05617978
Fibroblasts	negative regu	16-Mar	4.13E-05	0.0012467	0	0	55.3317887	558.579967	ISG15;SAMH	16	3	negative regu	0.1875
Fibroblasts	positive regu	20-Mar	8.30E-05	0.0020888	0	0	42.3040397	397.517861	DDX58;STAT1	20	3	positive regu	0.15
Fibroblasts	regulation of	23-Mar	0.00012774	0.00301393	0	0	35.9530121	322.336479	STAT1;PARP1	23	3	regulation of	0.13043478
Fibroblasts	positive regu	5-Feb	0.00018122	0.00390922	0	0	158.02381	1361.4995	HSPA1B;HSP	5	2	positive regu	0.4
Fibroblasts	vesicle fusio	6-Feb	0.00027107	0.00487287	0	0	118.511905	973.352544	TAP1;TAPBP	6	2	vesicle fusio	0.33333333
Fibroblasts	positive regu	Mar-32	0.00034791	0.0059698	0	0	24.7839634	197.368809	CD274;ISG1	32	3	positive regu	0.09375
Fibroblasts	regulation of	Apr-83	0.00045209	0.0069658	0	0	12.2476073	94.326662	ZBP1;IRF1;S	83	4	regulation of	0.04819277
Macrophages	cellular resp	15/65	1.53E-22	5.78E-20	0	0	83.9323944	4215.96131	IFITM3;SP10	65	15	cellular resp	0.23076923
Macrophages	response to i	28-May	1.19E-07	9.97E-06	0	0	53.3843264	851.257838	IFITM3;BST2;	28	5	response to i	0.17857143
Macrophages	interferon-ga	Jun-68	4.68E-07	3.21E-05	0	0	24.0145161	349.993722	SP100;STAT1;	68	6	interferon-ga	0.08823529
Macrophages	innate immu	10/302	6.83E-07	4.16E-05	0	0	8.84192502	125.527633	IFITM3;BST2;	302	10	innate immu	0.03311258
Macrophages	response to i	Jun-80	1.23E-06	5.82E-05	0	0	20.1081081	273.579583	IFITM3;BST2;	80	6	response to i	0.075
Macrophages	negative regu	May-54	3.50E-06	0.00015552	0	0	25.0251953	314.37249	IFITM3;BST2;	54	5	negative regu	0.09259259
Macrophages	response to t	9-Mar	6.33E-06	0.00026545	0	0	119.927711	1435.58847	SP100;ISG15	9	3	response to t	0.33333333
Macrophages	regulation of	May-64	8.16E-06	0.00030814	0	0	20.7731743	243.377286	IRF1;IFI35;PA	64	5	regulation of	0.078125
Macrophages	positive regu	9/335	1.38E-05	0.00047392	0	0	7.02302605	78.5887626	ZBP1;CD274	335	9	positive regu	0.02686567
Macrophages	positive regu	Apr-36	1.69E-05	0.00055467	0	0	30.3079268	333.034644	DDX58;IRF1;	36	4	positive regu	0.11111111
Macrophages	regulation of	May-89	4.08E-05	0.0012467	0	0	14.5723104	147.262113	ZBP1;DDX58	89	5	regulation of	0.05617978
Macrophages	negative regu	16-Mar	4.13E-05	0.0012467	0	0	55.3317887	558.579967	ISG15;SAMH	16	3	negative regu	0.1875
Macrophages	positive regu	20-Mar	8.30E-05	0.0020888	0	0	42.3040397	397.517861	DDX58;STAT1	20	3	positive regu	0.15
Macrophages	regulation of	23-Mar	0.00012774	0.00301393	0	0	35.9530121	322.336479	STAT1;PARP1	23	3	regulation of	0.13043478
Macrophages	positive regu	5-Feb	0.00018122	0.00390922	0	0	158.02381	1361.4995	HSPA1B;HSP	5	2	positive regu	0.4
Macrophages	vesicle fusio	6-Feb	0.00027107	0.00487287	0	0	118.511905	973.352544	TAP1;TAPBP	6	2	vesicle fusio	0.33333333
Macrophages	positive regu	Mar-32	0.00034791	0.0059698	0	0	24.7839634	197.368809	CD274;ISG1	32	3	positive regu	0.09375
Macrophages	regulation of	Apr-83	0.00045209	0.0069658	0	0	12.2476073	94.326662	ZBP1;IRF1;S	83	4	regulation of	0.04819277
Monocytes	SRP-depende	62/90	5.05E-89	1.07E-85	0	0	150.33391	30564.5895	RPL4;RPL5;R	90	62	SRP-depende	0.68888889
Monocytes	cellular resp	21/65	2.23E-21	2.64E-19	0	0	28.3543388	1348.30244	IFITM3;SP10	65	21	cellular resp	0.32307692
Monocytes	negative regu	14/54	3.50E-13	3.23E-11	0	0	20.3654303	584.126312	IFITM3;RSAD	54	14	negative regu	0.25925926

Monocytes	cellular resp	35/482	1.29E-12	1.10E-10	0	0	4.75795033	130.265373	IL1RN;CD40;	482	35	cellular resp	0.07261411
Monocytes	response to i	14/80	1.08E-10	7.90E-09	0	0	12.3263196	282.920001	IFITM3;GBP5	80	14	response to i	0.175
Monocytes	interferon-ga	13/68	1.58E-10	1.12E-08	0	0	13.7020979	309.244792	CAMK2D;SP1	68	13	interferon-ga	0.19117647
Monocytes	regulation of	Dec-64	1.02E-09	6.82E-08	0	0	13.3403676	276.128727	FGR;GBP5;IR	64	12	regulation of	0.1875
Monocytes	cellular resp	17/155	2.09E-09	1.35E-07	0	0	7.19619457	143.815916	IL1RN;CD40;	155	17	cellular resp	0.10967742
Monocytes	positive regul	Oct-45	4.51E-09	2.82E-07	0	0	16.4340176	315.815231	GBP5;GRN;C	45	10	positive regul	0.22222222
Monocytes	regulation of	Dec-83	2.24E-08	1.33E-06	0	0	9.76093731	171.92359	ZBP1;FGR;H	83	12	regulation of	0.14457831
Monocytes	positive regul	30/546	3.84E-08	2.21E-06	0	0	3.46536985	59.1747567	CD40;IFI35;D	546	30	positive regul	0.05494506
Monocytes	cellular resp	17/194	6.17E-08	3.46E-06	0	0	5.59937752	92.9532607	CD40;STAT1;	194	17	cellular resp	0.08762887
Monocytes	regulation of	Nov-74	6.48E-08	3.54E-06	0	0	10.0581699	166.482383	ZBP1;SOCS1	74	11	regulation of	0.14864865
Monocytes	regulation of	16/179	1.15E-07	5.97E-06	0	0	5.70966029	91.2282334	CD40;RSAD2	179	16	regulation of	0.08938548
Monocytes	positive regul	22/335	1.31E-07	6.66E-06	0	0	4.13094186	65.4531554	ZBP1;GBP5;C	335	22	positive regul	0.06567164
Monocytes	negative regu	16/182	1.45E-07	7.17E-06	0	0	5.6056105	88.275922	CD274;DDX5	182	16	negative regu	0.08791209
Monocytes	positive regul	Aug-36	1.64E-07	7.78E-06	0	0	16.3440233	255.371051	IFIH1;DDX58	36	8	positive regul	0.22222222
Monocytes	negative regu	16-Jun	1.93E-07	8.94E-06	0	0	34.1547826	528.004255	STAT2;OAS3;I	16	6	negative regu	0.375
Monocytes	innate immu	20/302	4.32E-07	1.96E-05	0	0	4.1496861	60.8124233	IFITM3;CD40	302	20	innate immu	0.06622517
Monocytes	regulation of	Nov-89	4.48E-07	1.99E-05	0	0	8.11768477	118.671104	IFIH1;ZBP1;D	89	11	regulation of	0.12359551
NK_cells	SRP-depende	62/90	5.05E-89	1.07E-85	0	0	150.33391	30564.5895	RPL4;RPL5;R	90	62	SRP-depende	0.68888889
NK_cells	cellular resp	21/65	2.23E-21	2.64E-19	0	0	28.3543388	1348.30244	IFITM3;SP10	65	21	cellular resp	0.32307692
NK_cells	negative regu	14/54	3.50E-13	3.23E-11	0	0	20.3654303	584.126312	IFITM3;RSAD	54	14	negative regu	0.25925926
NK_cells	cellular resp	35/482	1.29E-12	1.10E-10	0	0	4.75795033	130.265373	IL1RN;CD40;	482	35	cellular resp	0.07261411
NK_cells	response to i	14/80	1.08E-10	7.90E-09	0	0	12.3263196	282.920001	IFITM3;GBP5	80	14	response to i	0.175
NK_cells	interferon-ga	13/68	1.58E-10	1.12E-08	0	0	13.7020979	309.244792	CAMK2D;SP1	68	13	interferon-ga	0.19117647
NK_cells	regulation of	Dec-64	1.02E-09	6.82E-08	0	0	13.3403676	276.128727	FGR;GBP5;IR	64	12	regulation of	0.1875
NK_cells	cellular resp	17/155	2.09E-09	1.35E-07	0	0	7.19619457	143.815916	IL1RN;CD40;	155	17	cellular resp	0.10967742
NK_cells	positive regul	Oct-45	4.51E-09	2.82E-07	0	0	16.4340176	315.815231	GBP5;GRN;C	45	10	positive regul	0.22222222
NK_cells	regulation of	Dec-83	2.24E-08	1.33E-06	0	0	9.76093731	171.92359	ZBP1;FGR;H	83	12	regulation of	0.14457831
NK_cells	positive regul	30/546	3.84E-08	2.21E-06	0	0	3.46536985	59.1747567	CD40;IFI35;D	546	30	positive regul	0.05494506
NK_cells	cellular resp	17/194	6.17E-08	3.46E-06	0	0	5.59937752	92.9532607	CD40;STAT1;	194	17	cellular resp	0.08762887
NK_cells	regulation of	Nov-74	6.48E-08	3.54E-06	0	0	10.0581699	166.482383	ZBP1;SOCS1	74	11	regulation of	0.14864865
NK_cells	regulation of	16/179	1.15E-07	5.97E-06	0	0	5.70966029	91.2282334	CD40;RSAD2	179	16	regulation of	0.08938548
NK_cells	positive regul	22/335	1.31E-07	6.66E-06	0	0	4.13094186	65.4531554	ZBP1;GBP5;C	335	22	positive regul	0.06567164
NK_cells	negative regu	16/182	1.45E-07	7.17E-06	0	0	5.6056105	88.275922	CD274;DDX5	182	16	negative regu	0.08791209
NK_cells	positive regul	Aug-36	1.64E-07	7.78E-06	0	0	16.3440233	255.371051	IFIH1;DDX58	36	8	positive regul	0.22222222
NK_cells	negative regu	16-Jun	1.93E-07	8.94E-06	0	0	34.1547826	528.004255	STAT2;OAS3;I	16	6	negative regu	0.375
NK_cells	innate immu	20/302	4.32E-07	1.96E-05	0	0	4.1496861	60.8124233	IFITM3;CD40	302	20	innate immu	0.06622517
NK_cells	regulation of	Nov-89	4.48E-07	1.99E-05	0	0	8.11768477	118.671104	IFIH1;ZBP1;D	89	11	regulation of	0.12359551
Neutrophils	SRP-depende	62/90	5.05E-89	1.07E-85	0	0	150.33391	30564.5895	RPL4;RPL5;R	90	62	SRP-depende	0.68888889
Neutrophils	cellular resp	21/65	2.23E-21	2.64E-19	0	0	28.3543388	1348.30244	IFITM3;SP10	65	21	cellular resp	0.32307692
Neutrophils	negative regu	14/54	3.50E-13	3.23E-11	0	0	20.3654303	584.126312	IFITM3;RSAD	54	14	negative regu	0.25925926
Neutrophils	cellular resp	35/482	1.29E-12	1.10E-10	0	0	4.75795033	130.265373	IL1RN;CD40;	482	35	cellular resp	0.07261411
Neutrophils	response to i	14/80	1.08E-10	7.90E-09	0	0	12.3263196	282.920001	IFITM3;GBP5	80	14	response to i	0.175
Neutrophils	interferon-ga	13/68	1.58E-10	1.12E-08	0	0	13.7020979	309.244792	CAMK2D;SP1	68	13	interferon-ga	0.19117647
Neutrophils	regulation of	Dec-64	1.02E-09	6.82E-08	0	0	13.3403676	276.128727	FGR;GBP5;IR	64	12	regulation of	0.1875
Neutrophils	cellular resp	17/155	2.09E-09	1.35E-07	0	0	7.19619457	143.815916	IL1RN;CD40;	155	17	cellular resp	0.10967742
Neutrophils	positive regul	Oct-45	4.51E-09	2.82E-07	0	0	16.4340176	315.815231	GBP5;GRN;C	45	10	positive regul	0.22222222
Neutrophils	regulation of	Dec-83	2.24E-08	1.33E-06	0	0	9.76093731	171.92359	ZBP1;FGR;H	83	12	regulation of	0.14457831
Neutrophils	positive regul	30/546	3.84E-08	2.21E-06	0	0	3.46536985	59.1747567	CD40;IFI35;D	546	30	positive regul	0.05494506
Neutrophils	cellular resp	17/194	6.17E-08	3.46E-06	0	0	5.59937752	92.9532607	CD40;STAT1;	194	17	cellular resp	0.08762887
Neutrophils	regulation of	Nov-74	6.48E-08	3.54E-06	0	0	10.0581699	166.482383	ZBP1;SOCS1	74	11	regulation of	0.14864865
Neutrophils	regulation of	16/179	1.15E-07	5.97E-06	0	0	5.70966029	91.2282334	CD40;RSAD2	179	16	regulation of	0.08938548
Neutrophils	positive regul	22/335	1.31E-07	6.66E-06	0	0	4.13094186	65.4531554	ZBP1;GBP5;C	335	22	positive regul	0.06567164
Neutrophils	negative regu	16/182	1.45E-07	7.17E-06	0	0	5.6056105	88.275922	CD274;DDX5	182	16	negative regu	0.08791209
Neutrophils	positive regul	Aug-36	1.64E-07	7.78E-06	0	0	16.3440233	255.371051	IFIH1;DDX58	36	8	positive regul	0.22222222
Neutrophils	negative regu	16-Jun	1.93E-07	8.94E-06	0	0	34.1547826	528.004255	STAT2;OAS3;I	16	6	negative regu	0.375
Neutrophils	innate immu	20/302	4.32E-07	1.96E-05	0	0	4.1496861	60.8124233	IFITM3;CD40	302	20	innate immu	0.06622517
Neutrophils	regulation of	Nov-89	4.48E-07	1.99E-05	0	0	8.11768477	118.671104	IFIH1;ZBP1;D	89	11	regulation of	0.12359551
Proliferating	mitochondri	2/175	0.00074818	0.01846831	0	0	76.3853565	549.811386	BNIP3L;COX	175	2	mitochondri	0.01142857
T_CD4	mitochondri	2/175	0.00074818	0.01846831	0	0	76.3853565	549.811386	BNIP3L;COX	175	2	mitochondri	0.01142857
T_CD8	mitochondri	2/175	0.00074818	0.01846831	0	0	76.3853565	549.811386	BNIP3L;COX	175	2	mitochondri	0.01142857
