# Supplementary material for: Redundant and distinct mechanisms suppress innate immune activation during SARS-CoV-2 infection
Source: PLoS Biol. 2026 May 20;24(5):e3003808. doi: 10.1371/journal.pbio.3003808 (PMC13221149; doi:10.1371/journal.pbio.3003808)
Supplement: S12 Fig — Activation of DC subpopulations characterized by single-cell sequencing. A. Integrated re-clustered UMAP of all mock (n = 3), WT (n = 4), NSP1 mutant (n = 4), and NSP15 mutant (n = 4) cells in the DC population colored by cluster number (top) and DC subtype (bottom, based on markers in panel C). B. Dot plot showing canonical marker expression in the DC sub-clusters. Relevant marker sets for each DC sub-population are highlighted in the same color as the corresponding sub-populations in the UMAP (B, bottom). C. Box plots comparing cell-type proportions of the DC sub-populations observed in Mock, WT, NSP1, and NSP15 groups. The limits of the box reflect the interquartile range (IQR: Q3–Q1) with the medians shown as horizontal bars. Whiskers extend to 1.5 times the IQR of the box. For each cell type, pairwise t test comparisons of the wild type (WT) proportion with every other group are shown (*p-value < 0.05, ***p-value < 0.001, ns - nonsignificant). The data underlying this Figure can be found in GEO database, accession number GSE 255483. (PDF) [file pbio.3003808.s012.pdf]

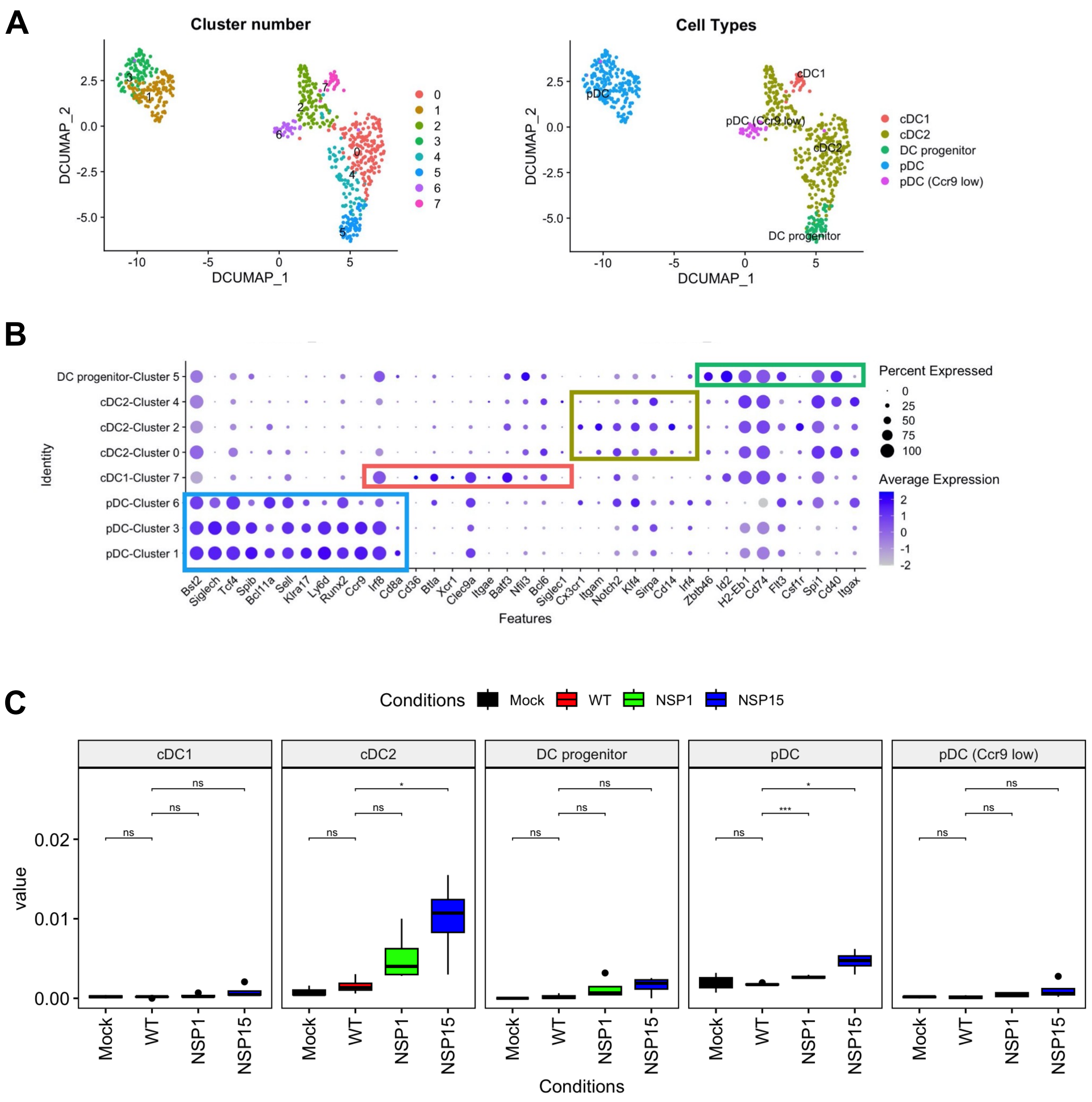

Suppl. Fig. 12. Activation of DC subpopulations characterized by single-cell sequencing.
A. Integrated re-clustered UMAP of all mock (n = 3), WT (n = 4), NSP1 mutant (n = 4), and NSP15 mutant (n = 4) cells in the DC population colored by cluster number (top) and DC subtype (bottom, based on markers in panel C).
B. Dot plot showing canonical marker expression in the DC sub-clusters. Relevant marker sets for each DC sub-population are highlighted in the same color as the corresponding sub-populations in the UMAP (B, bottom).
C. Box plots comparing cell-type proportions of the DC sub-populations observed in Mock, WT, NSP1, and NSP15 groups. The limits of the box reflect the interquartile range (IQR: Q3–Q1) with the medians shown as horizontal bars. Whiskers extend to 1.5 times the IQR of the box. For each cell type, pairwise t-test comparisons of the wild type (WT) proportion with every other group are shown (* p-value < 0.05, *** p-value < 0.001, ns - non-significant).
The data underlying this Figure can be found in GEO database, accession number GSE 255483.
